# Acknowledgment to the Reviewers of *Life* in 2022

**DOI:** 10.3390/life13020260

**Published:** 2023-01-17

**Authors:** Life Editorial Office

**Affiliations:** MDPI AG, St. Alban-Anlage 66, 4052 Basel, Switzerland

High-quality academic publishing is built on rigorous peer review. *Life* was able to uphold its high standards for published papers due to the outstanding efforts of our re-viewers. Thanks to the efforts of our reviewers in 2022, the median time to first decision was 13.4 days and the median time to publication was 34 days. Regardless of whether the articles they examined were ultimately published, the editors would like to express their appreciation and thank the following reviewers for the time and dedication that they have shown *Life*:
A. GanesanKyle R. BohnertA. N. M. Mamun-Or-RashidKyoung-Chan ParkA. S. LukatkinKyung Hwan KimAalap ChokshiKyung Rae KoAaron ManzanaresKyung Sik ParkAaron Silva SanchezKyunghoon MinAarti BainsKyung-Min KimAbazar GhorbaniKyung-Sook ChoiAbbas F. AlmullaKyung-Yong ChungAbbas JarrahiKyuseok KimAbbas RezaeeL. Drake DemingAbbas SmileyLadislav MicaAbbu ZaidLafon-Hughes LauraAbdelfattah El MoussaouiLakshmi PrabhuAbdelfattah M. SelimLalit BatraAbdelghafar Abu-ElsaoudLamei LeiAbdelmalek BouguettayaLamiaa M. A. AliAbderrahmen MerghniLampros FotisAbdol PaghehLara CarvalhoAbdolrazagh Hashemi ShahrakiLara HilalAbdul Awal Chowdhury MasudLara RighiAbdulkadir AtalanLarisa RyskalinAbdülkadir TunçLarissa FernandesAbeer Rababa'HLarissa StrathAbhay B. FulkeLars NorgrenAbhijit A. GhadgeLars P. KamolzAbhijit ChakrabartiLászló BozóAbhinav JainLászló RátgéberAbhinava K. MishraLata SinghAbhishek ChauhanLaura FumagalliAbhishesh MehataLaura GiganteAbid AliLaura Grațiela VicașAbid HussainLaura MaziluAbid UllahLaura MitreaAbigail Morales-SánchezLaura SalvadoriAbirami Ramu GanesanLaura SantagostiniAbo Elsoud Mostafa M.Laurel O. SillerudAbooali GolzaryLauren DoumaAbouzar NajafiLauren R. CirrincioneAbraham Loera MuroLaurent BonnacAcga ChengLaurent MetzingerAchyut TiwariLaurent PlantierAdalberto Alves Pereira-FilhoLaurent SebbagAdam AbiedLaurent VuatazAdam KernLazar B. DavidovicAdam OkorskiLazaros TzelvesAdam ReichLeandro MamoneAddy ProssLee Seong WeiAdela BadauLeela KaurAdelaida AvinoLei ChangAdeyemi Oladapo AremuLei LiuAdhimoolam KarthikeyanLei WangAdil AsgharLei ZhouAdina HutanuLeila GironAditi KulkarniLena MöbusAditi SinghviLenka KlčováAdolfo R. CalorLenka MichalcovaAdrià ArboixLennart NilssonAdrian Florin GalLeonardo Alexandre Peyré-TartarugaAdrian HarrisonLeonardo BrunettiAdrian NeaguLeonardo CentonzeAdrian PatersonLeonid BystrykhAdrián Varela SanzLeonid Pavlovich ChurilovAdriana AlbuLeopoldo FornerAdriana BalanLeslie BuckAdriana GrigorașLeslie Chavez-GalanAdriana HanganLesly Tejada BenitezAdriana TrifanLeszek BlicharzAdriano DuattiLeszek SieczkoAdrie VerhoevenLeszek TylickiAfshan MasoodLetizia Corinna MorlacchiAftab Ali ShahLi XiaoAftab BashirLiang HuAftab YaseenLiang PeiAfzal MisraniLiang Ting LinAgata GabryelskaLiang YeAgata GóźdźLiang-Jun YanAgata Sobczyńska-MaleforaLiborija Lugovic-MihicAgata TyczewskaLiborija Lugović-MihićAgata ZdartaLida FekratAgata ZnamirowskaLidia BłaszczykAglaia PappaLidia Chitimia-DoblerAgnes FleuryLiew Hon JungAgnes JanovszkyLigia DutuAgnes KlarLi-Han ChenAgnese De MarioLihong ZhaiAgnieszka KamińskaLihua ZhaoAgnieszka KwiatekLijing XinAgnieszka OrkuszLijun WeiAgnieszka Owczarczyk-SaczonekLilach GavishAgnieszka Synowiec-WojtarowiczLiliana CepoiAgnieszka Ulatowska-JarżaLiliana Mititelu TartauAgostino GuidaLiming WuAgre PaterneLin JiangAgustín Aibar-AlmazánLinbo ZhaoAgustinus Purna IrawanLinda HersheyAhmad AljaghsiLindell K. WeaverAhmad ArzaniLingyi LiuAhmad CharifaLinjun HongAhmad FarazLinto ThomasAhmar RaufLinxia GuAhmed A. ZakyLinyuan MaAhmed Abdelbaset-IsmailLiping GuAhmed Edan DhamadLisa LandgrafAhmed ElamragyLisa SchülerAhmed ElbestawyLisanne De BoerAhmed ElmassryLi‐Ting MaAhmed GalhoumLiubov OsminkinaAhmed KhalifaLiudmila Gerasimova-MeigalAhmed MahalLiuqi GuAhmed NafisLivia KapustaAhmed S. MandourLivio VitielloAhmed ShehabLjubisa NikolicAhmed TurhanLoganathan RangasamyAhmet AltindagLoh Teng-Hern TanAhmet Ercan TopcuLong ChenAhmet ÖzLonnie Van ZylÁislan De C. VivariniLoredana BergandiAistė BulavaitėLoredana CiarmielloAiyou HuangLoredana Elena VijanAjaikumar KunnumakkaraLorella PaparoAjar Nath YadavLorena Aguilar-ArnalAjay KumarLorenz AglasAjay SinghLorenz LeitnerAjaz AhmadLorenzo FaggioniAji Arif SabtaLorenzo Ferro DesideriAjith PolonowitaLorys CastelliAjmer Singh GrewalLouis S. H. LeeAkalesh Kumar VermaLourdes Cortes-DericksAkbar MohammadLuan Luong ChuAkhil VarshneyLuana PerioliAkhilesh PathakLuca BrazziAkihiko HoshiLuca CerritelliAkihiro OhsumiLuca CostanzoAkihisa TakahashiLuca Di GianfrancescoAkiko SekiguchiLuca GiacomelliAkiko SuzukiLuca Giovanni LocatelloAkio OishiLuca Lo PiccoloAkira TeramotoLuca LombardoAkito NakagawaLuca MenilliAkiyoshi OginoLuca MiceliAkriti SrivastavaLuca Oscar Redaelli De ZinisAlain ArtolaLuca Salvatore De SantoAlain RamanantsoanirinaLuca UrsoAlan PalmerLucia CarboniAlarico ArianiLucia NastiAlba CorellLucia StančiakováAlba Ester IlleraLucia Vieira HoffmannAlba Marina GimenezLucian CopoloviciAlbert RubbenLucian NegreanuAlbert S. Y. WongLuciana BordinAlbert SabbanLuciana Pereira XavierAlberto A. Escobar-PuentesLucie MarousezAlberto ChighineLuciene Da Cruz FernandesAlberto FantinŁucja PrzysieckaAlberto Lo GulloLudmiła Daniłowicz‑SzymanowiczAlberto MellaLudmila KazdovaAlberto MiceliLudmila N. BakhirevaAlbrecht OttLudovico MessineoAldo Alvarez-RiscoLuigi Alberto PiniAldo ClericoLuigi BattistaAldo CombarizaLuigi BennardoAldo Proietti-JuniorLuigi CoppolaAlejandro Heredia BarberoLuigi Della CorteAlejandro Schcolnik-CabreraLuigi Di TommasoAlejandro Suarez-De-La-RicaLuigi DonatoAlejandro ValbuenaLuigi MeccarielloAlejandro VallejoLuigi-Alberto PiniAleksander F. SikorskiLuis AlmeidaAleksander ZwierzLuis Antônio EsmerinoAleksandra Karczmarska-WódzkaLuis Castruita-EsparzaAleksandra KristoLuis Ceballos-LaitaAleksandra MaršavelskiLuis DelayeAleksandra RanitovićLuis Exequiel IbarraAleksandra SavićLuis L. BonillaAleksandra SknepnekLuis MontenegroAleksandra SzczepankiewiczLuis Roberto De Camargo GonçalvesAleksandra SzczepkowskaLuis RodrigoAleksandra SzopaLuisa ArrudaAleksandra UzelacLuisa RicciardiAleksandra ZeljkovicLuiz CarneiroAleksei KholodovLuiz Gustavo De Almeida ChuffaAleksei ZverevLuiz Gustavo Dos Anjos BorgesAleksey ChaulinLuiza Marek-JózefowiczAlena FurdovaLukáš SmolkoAleš PavlíkŁukasz KaczmarekAlessandra BosuttiLukasz LapajAlessandra D’AmicoŁukasz M. MilanowskiAlessandra FilosaŁukasz SobottaAlessandra LaforgiaLuke ChongAlessandra MarconiLuke HeemsbergenAlessandra SinopoliLuke PaterAlessandro De SireLulin HuangAlessandro DelitalaLumei PuAlessandro MalobertiLura LlullAlessandro MeduriLu-Sheng HsiehAlessandro PalleschiLuz Breton-DevalAlessandro PerrellaLuz Kelly AnzolaAlessandro TerrinoniM. ArulperumjothiAlessandro ViganòM. F. NadeemAlessandro VittoriM. R. MohammadabadiAlessandro ZucchiMa Cristina Del Rincón-CastroAlessia CarboniMaan RokayaAlessia CatalanoMaarten VanhaverbekeAlessia FilipponeMaciej CedzyńskiAlessio AlesciMaciej ChęcińskiAlessio FacchinMaciej GawęckiAlessio PaladiniMaciej KamaszewskiAlessio RocconMaciej SikoraAlex Buoite StellaMaciej SkrzypczakAlex ComptonMacrina B. Silva-CázaresAlex M. ValmMaddalena ParafatiAlexander A. SpasovMaddeboina KrishnaiahAlexander A. StakheevMadhan JeyaramanAlexander BerezinMadhulika PradhanAlexander E. KabakovMaedeh GhasemiAlexander LarinMafatlal M. KherAlexander O. ChizhovMafumi HishidaAlexander OtahalMagda Latorre-EstevezAlexander RufMagdalena Czarnecka-OperaczAlexander SimakinMagdalena E. Paweł'KowiczAlexander V. NosovMagdalena Frej-MądrzakAlexander VoronkovMagdalena KostrzewaAlexander ZaboronokMagdalena Krajewska-WłodarczykAlexander ZhgunMagdalena Mackiewicz-MilewskaAlexandra BurluiMagdalena PiekutowskaAlexandra KalogerakiMaged S. Abdel-KaderAlexandra MartuMagno Conceição Das MercêsAlexandra-Dana ChițimusMaha FadelAlexandre BergantiniMahammed MoniruzzamanAlexandre G. De BrevernMahboob AlamAlexandros GeorgakilasMahboobeh HodaeiAlexandru AchimMahdi MahdipourAlexandru BejinariuMahdieh MehrpouriAlexandru BurlacuMahi M. MohiuddinAlexandru CorlateanuMahipal Singh KesawatAlexandru UliciMahmoud A. Al-SamanAlexandru Vasile RusuMahmoud A. SenousyAlexandru VlaicuMahmoud Abo-AshourAlexey A. KytmanovMahmoud AzzamAlexey LipengoltsMahmoud DiabAlexey MeshkovMahmoud I. KhalilAlexey SuminMaicol ManciniAlexey V. FeofanovMailo VirtoAlexis StamatikosMaite VaslinAlexsandra Fernandes PereiraMaja BaretićAlfonso Martínez-NovaMaja CupicAlfonso Penichet-TomásMaja MatulićAlfredo CaturanoMaja ŠantakAlfredo CiccodicolaMajed H. WakidAlfredo ContiMajid KomijaniAlfredo IandoloMajid KoozehchianAli BaghdadiMajid MohhamadhosseiniAli BoolaniMajid Sharifi RadAli Kerim YılmazMakoto FurugenAli KrztonMakoto HasegawaAli RostamiMakoto HashimotoAli ZarrabiMakoto NakamuraAlicia Gamboa-DebuenMakoto TakeiAlicia Negrón MendozaMaksymilian ProndzynskiAlicia Romero-LorcaMaleeya KruatrachueAlicja BorońMalgorzata BurekAlina AlexandrovaMałgorzata IciekAline Maria Da SilvaMalgorzata KrawczykAlireza AhmadianMalgorzata MikiuciukAlireza KarimiMałgorzata Polz-DacewiczAlireza MardomiMałgorzata ŚwiątkiewiczAlireza RafieiMałgorzata WojtyśAlireza ZarebidokiMałgorzata WrzosekAliza Toby EhrlichMalgorzata Zakłos-SzydaAlma Delia Genis-MendozaMalik SajidAlok MondalMalik SallamAlok SutradharMalte BraackAlon SavidorMalvin N. JanalAlp SenerMamdouh EissaAlper TokerMami KayamaAlvaro CrevennaMamoona HumayunÁlvaro F. FernándezMamoru TakedaAlvaro MaciasManal FawzyAlvaro PérezManasvi LingamAlvise SernicolaMandana KamgarAlyssa M. WeinrauchManisekaran RavichandranAmalendu GhoshManish RaturiAman KarimManish SharmaAmanda IglesiasManish Vijaya KumarAmany MohamedManisha BanerjeeAmany SholkamyManjeet SinghAmar KrishnaManoj KumarAmar Nath ChatterjeeManoj Kumar TripathiAmarinder SinghManolis WallaceAmber DoreyManpreet S. BhattiAmeesh M. IsathManu KumarAmela JusicManuel Gomez-BarreraAmelia BarcelliniManuel Hermida PrietoAmelia Portillo LópezManuel Nieto-DiazAmélie GabetManuel Prado-VelascoAmgad AlbohyManuel Rebollo-SalasAmila AkagicManuel RequenaAmin R. JaverManuel Sanchez-DiazAmir FaisalManuel ValdebranAmir HadiManuela Reyes-EstebanezAmirah GozaliMaohua YangAmirhossein AhmadiMaqsood MalikAmir-Houshang ShemiraniMara CarsoteAmit AgrawalMarawan AbdelbasetAmit Baran SharangiMarc FolcherAmit GuptaMarc G. J. FeuiloleyAmit KumarMarc MarescaAmit Kumar MishraMarcel LemireAmit Kumar Singh GautamMarcela FrankovaAmit SrivastavaMarcela PaganoAmiya PatraMarcello AugelloAmmad Ahmad FarooqiMarcello CasertanoAmmar AltemimiMarcello MaestriAmmar BaderMarcello RomanoAmnah Mohammed AlsuhaibaniMarcelo ArancibiaAmosy M'KomaMarcelo DavidAmr Ahmed El‑ArabeyMarcelo Maia PereiraAmr FoudaMarcelo Pires PradoAmr Mohamed Samy MahdyMarcin ArciszewskiAmritlal MandalMarcin BanachAmzad HossainMarcin DerwichAna BorotaMarcin DrozdAna C. GonçalvesMarcin DziekiewiczAna Čipak GašparovićMarcin FeldoAna Clara AprotosoaieMarcin GierekAna Cristina Breithaupt-FaloppaMarcin KidońAna CusumanoMarcin KucińskiAna DascaluMarcin OkrójAna DobrevaMarcin PorebaAna I. Álvarez-MercadoMarcin RozalskiAna I. Benítez-MateosMarcin ZielinskiAna IvaniševićMarcio DornAna Karla M. LoboMarco A. RamosAna Lilia Torres-MachorroMarco ArticoAna Luísa De Sousa-CoelhoMarco BrunoriAna Luisa Garcia OliveiraMarco C. CavacoAna Marta De MatosMarco CamperaAna RochaMarco CicconeAna Sanchez-RodriguezMarco CoppiAna SilvaMarco CucurulloAna Sofia DuarteMarco Di BattistaAna TiganescuMarco DolciAna TomićMarco FerrariAna UmpiérrezMarco FoganteAnabela CorreiaMarco FracassettiAnagha KrishnanMarco JoseAnahita Jablonski-MomeniMarco PortelliAnalìa L. ArévaloMarco PrenassiAna-Maria GalanMarco SapienzaAnan JongkaewwattanaMarco SoligoAnan KenthaoMarco Stefano DemarchiAnand KumarMarco TafaniAnand MaryaMarcos Eric Barbosa BritoAnand Prakash SinghMarcus D. LanceAnand SharmaMarcus FelicianoAnand SinghMarcus GraneggerAnas RaklamiMarcus LancéAnas ShamsiMarcus ScottiAnastas GospodinovMargarida Pedroso De LimaAnastasia HyrinaMargarita IvanovaAnastasia MalekMargherita EufemiAnastasia SpiliopoulouMaria A. MariggiòAnatoly SkripalMaria Al-MatarnehAnbarasu KannanMaria AndreoliAnca UngurianuMaria Angeles RojoAnca-Roxana PetroviciMaria Borszewska-KornackaAnchalee ChurojanaMaria Carmen MolinaAnders KjellbergMaría Colín-GarcíaAndi AlijagićMaria Cristina De RosaAndi Dian PermanaMaria Cristina SorrentinoAndoni Carrasco-UribarrenMaria De La Paz SánchezAndrada PicaMaría Del Carmen García-RíosAndras BikovMaría Del Pilar CordovillaAndrás BikovMaría Dolores Cima-CabalAndre BrackMaría Elizbeth Alvarez-SanchezAndré BrackMaria Fátima PaulinoAndre Luiz CostaMaria FortofoiuAndré Mariz De AlmeidaMaria GiordanoAndré P. WolffMaria Giovanna ScioliAndré Rolim BabyMaria Grazia BottoneAndré WilkeMaria Grazia Di CertoAndrea AbateMaria Helena S. GoldmanAndrea AngeliniMaría Inés RagoneAndrea BerenyiovaMaría Jesús Alvarez CuberoAndrea BuonoMaria Jose BelliniAndrea ButeraMaría José BessoAndrea D'AvieroMaría José Ruiz-MagañaAndrea DesideratoMaria KapritsouAndrea L. HildebrandMaría Laura PeschiuttaAndrea MagrìMaria Magdalena VasilescuAndrea Milostić SrbMaria MaistoAndrea PiccioniMaria ManiAndrea PoloMaría Margarita Canales-MartínezAndrea RagusaMaria MoschoviAndrea Roxanne J. AnasMaria OczkowiczAndrea SambriMaria PaparoupaAndrea V. PeraltaMaria PerdomoAndrea Vukić DugacMaria Pokorska-ŚpiewakAndreas MerdesMaría Prado-NovoaAndreas PetryMaria Romerowicz-MisielakAndreas RitterMaria Rosaria BarillariAndreas S. PapazoglouMaria Rosaria De MiglioAndreea Catarina PopescuMaria Rosaria RuscianoAndrei AlyokhinMaría Salazar-RoaAndrei I. KhlebnikovMaría SerramitoAndrei S. RodinMaria ShabbirAndrej KastrinMaria SimonenkoAndrés De La EscosuraMaria SpletterAndrés G. Vidal-GadeaMaria TalmonAndrés Juan KreinerMaria Teresa CeccheriniAndrés Molero ChamizoMaria Teresa PaganoAndrêssa Silva FernandesMaria TotanAndrew J. KobetsMaria V. MedvedevaAndrew LavenderMaria VaccaroAndrew LeaskMaríA Verónica BaezAndrew LeeMaria Vittoria CubellisAndrew NewbergMaria WróbelAndrew NorrisMaria ZanklAndrew OzgaMaría-Dolores ReyAndrew R. BurgoyneMarialuigia FantacuzziAndrey KuzminMarian SimkaAndrey PlotnikovMariana CamargoAndrey SimbirtsevMariana FloriaAndrija MatetićMariana María HuertaAndrius PranskunasMariana MaronezeAndrzej KasperskiMariana Pereira AntoniassiAndrzej KlasaMariangela AgamennoneAndrzej O. BieńkowskiMariangela ManciniAndrzej PolańczykMariángeles DíazAndy (Xiaoyu) WangMariann HarangiAndy Wai Kan YeungMarianne BurbageAneela Zameer DurraniMariano Francesco CaratozzoloAnelia IantchevaMariano VenanziAneliya MilanovaMariany Costa DepráAneta Agnieszka BartosikMarie MortreuxAnette KaiserMarie-Cécile NassogneAngel Guillermo BracamonteMarie-Christine MaurelAngel León-BuitimeaMariel CoradinÁngel Ricardo PlastinoMarika PellegriniAngela BaldanzaMariko OkamotoAngela Bernabeu SanzMarilena OneteAngela CapocefaloMarilena RonzanAngela TramontiMarilia CarabottiAngelica GiulianiMarilisa Pia DimmitoAngelica Lodin-SundströmMarimar M. Saez De Ocariz-GutierrezAngelika Tkaczyk-WlizłoMarina Consuelo VitaleAngelika V. TimofeevaMarina HolyavkaAngeliki AngelidiMarina KarpenkoAngelo Di IorioMarina P. IsaevaAngelo NaselliMarina PadilhaAngelo TorrenteMarina PadkinaAnggit SunarwidhiMarine CouéAngus YuMarinela BostanAniela PopescuMarino PrearoAniello MaieseMarino VilovićAnil Kumar ChallaMario GiuffridaAnindit MukherjeeMario GorenjakAnis ShahMario SimirgiotisAnish K. RayMariola KrodkiewskaAnish ShivaramMarion EhrichAnissa ChouikhaMarios SpanakisAnisuz ZamanMaristella MastoreAnita GuptaMarius RademakerAnita Lyssek-BorońMariusz DubielAnja KarlstaedtMariusz KoniecznyAnja Kathrin JaehneMariusz SikoraAnja KolaričMariusz UchmanAnju R. BabuMariya A. SmetaninaAnkit ManglaMariya V. EdelevaAnkit SrivastavaMarjan SabbaghianAnkita PunethaMarjan VračkoAnn Helen GaustadMarjorie PionAnn LiebertMárk AntalAnn SvenssonMark BelkAnna Bednarska-CzerwińskaMark H. BilskyAnna BizońMark KeithAnna BrzeckaMark S. ScherAnna Cleta CroceMark S. TaylorAnna HonkanenMarko KrstićAnna Jakubska-BusseMarko KumrićAnna Kostecka-GugalaMarkus SchoenbergAnna Machoy-MokrzyńskaMarleide Da Mota GomesAnna Makuch-KockaMarlid Cruz-RamosAnna Maria AloisiMarny FedrigoAnna Maria CostaMarta AltieriAnna Maria KotMarta CzaplickaAnna PerriMarta Del BiancoAnna PodlasekMarta Diaz-DelcastilloAnna SerefkoMarta Elena Losa IglesiasAnna ShcheglovaMarta Gonzalez-AlvarezAnna SobiepanekMarta KopańskaAnna SperanskayaMarta MuszalikAnna StarzyńskaMarta Nowacka-ChmielewskaAnna ValentinoMarta RybskaAnna ViolaMarta TomczykAnna WrzosekMartha Patricia Gallegos-ArreolaAnna Zalewska-JanowskaMartha-Spyridoula KatsarouAnnarita NappiMartin A. EstermannAnne Balkema-BuschmannMartin A. SchroerAnne FalcouMartin BartasAnne GuertlerMartin KelloAnne JörnsMartin P. PlayfordAnne-Sophie WoznyMartin PéčAnnette PfahlbergMartín RamírezAnn-Kristin Östlund-FarrantsMartin RasporAnoop AlexMartin TeraaAnoop Kumar SrivastavaMartina Baiardo RedaelliAnroop NairMartina BednářováAntanas VaitkusMartina Dal BelloAntero J. AbrunhosaMartina IrionAntje Büttner-TeleagăMartina MaggiAnton SdobnovMartina RossiAntonella AmicucciMartina TorricelliAntonella MuscellaMarvin A. Soriano-UrsúaAntonella MutoMarvin Antonio Soriano-UrsuaAntonella RosiMarwa A. A. FayedAntonella SmeriglioMarwa Mohamed ZalatAntonello SantiniMarwan El MobadderAntonia CianciulliMary BoardAntonija TadinMary Katherine MillsAntonino ManiaciMary Kearns-JonkerAntonio BrunoMary M. Y. WayeAntonio Carlos Silva-FilhoMaryam ArdalanAntonio CicioneMaryam AzlanAntonio Córdoba FernándezMaryam NakhjavaniAntonio Diogo Silva VieiraMaryam PoursadeghfardAntonio FacciorussoMarzia CottiniAntonio G. SolimandoMarzia OgnibeneAntonio García AbrilMarzia SegùAntonio GiordanoMaša PintaričAntonio Giovanni SolimandoMaša Skelin KlemenAntonio Giuseppe BiondiMasaaki HigashinoAntonio GonzàLez JuanMasafumi FunamotoAntonio José Piantino FerreiraMasafumi MurataniAntonio LeoMasahiko SugimotoAntonio MazzellaMasakazu MinetamaAntonio NennaMasakazu TanakaAntonio OrlacchioMasato KantakeAntonio Piñero-MadronaMasayoshi ShinjohAntonio PontorieroMasayuki ShimaAntonio Popolo RubbioMasfueh RazaliAntonio R. VieiraMasindi MphaphathiAntonio RizzaMasoud KeikhaAntonio Rodriguez-SinovasMassimiliano CadamuroAntonio Simone LaganàMassimiliano ChettaAntonio TrovatoMassimiliano De PaolisAntonio Victor Campos CoelhoMassimiliano RenziAntony AnanthMassimo BusinAnu Anna GeorgeMassimo CapocciaAnuj KumarMassimo ChelloAnupam KumarMassimo Di GiulioAnupam MukherjeeMassimo GuerrieroAnuphap PrachumwatMassimo TacchiniAnuraag BoddupalliMassimo TrottaAnurag MalikMassimo TusconiAnusak KerdsinMateus R. AmorimAnutosh PariaMateusz KowalAnzhelika VorobyevaMathew W. MaccumberAo JiangMathieu LetellierApichat VittaMathieu PicardeauApostolos BeloukasMatías Gastón PérezAqel AlbuttiMatonickjn Kepcija RenataAracely Serrano-MedinaMatteo Bruno LodiArafat Abdel Hamed Abdel LatefMatteo De MarcoArakere Chunchegowda UdayashankarMatteo FontanaAranzazu ManzanoMatteo MontanariArcangelo LisoMatteo PonzoniArchita Venugopal MenonMatteo RiccòArghadip SamaddarMatthew ButlerArif KhanMatthew GageArif MermerMatthias GatzArif Sabta AjiMatthias NéesArif Tasleem JanMatthias OckerArijit A. AdhikariMatthias PortArindam PramanikMatthias RiessArjen CupidoMatthieu Pierre PlatreArkaitz MucientesMattias AnderssonArman ArabMau-Ern PohArmando Vega-LoṕezMaurizio BadianiArmen MulkidjanianMaurizio FossarelloArnab GhoshMaurizio NordioArnaud BianchiMaurizio PiergiovanniArnaud LemmersMauro AlaibacArnaud PallottaMauro Cesar Palmeira VilarArne KienzleMauro GiuffrèAroub LahhamMauro LombardoArpita RoyMauro PistelloArrigo CiceroMauro ValenteArsen S. MikaelyanMax SarmetArtem P. LisachovMaxime RobinArthur FerreiraMaxime TourteArtur KrzyżakMaya M. ZaharievaArtur PasternakMaya PetekArtur SłomkaMayra MejíaArturo Cortés-TéllesMayumi NishiAruliah RajasekarMayur B. KaleArumugam RajaveluMayuren CandasamyAruna AmarasingheMayya RazgonovaArutselvan NatarajanMazhar Javed AwanArvind GulbakeMd MohiuddinArvind Kumar DubeyMd Robiul IslamArvind NegiMd Tahjib-Ul-ArifAsher FawwadMeenakshi S. KagdaAshim GuptaMeenu GuptaAshish AgrawalMeerambika MishraAshish NoronhaMegan RombergAshish RunthalaMehdi AjorlooAshkan ZandiMehdi Nasr IsfahaniAshok KumarMehmet Ali SaridaşAshraf Al-BrakatiMehmet AydinAshraf KariminikMehmet GülAshraf VirmaniMehmet YamanAsif AliMehrdad DadgostarAsif KhaliqMehtap SahinerAsif RazaMeixue DongAsim Kumar BepariMeiyan WangAsker KhapchaevMeliha EkinciAşkım Hediye SekmenMelinda MadlénaAslı Aksoy GündoğduMelissa MarianaAsli YilmazMenake PiyasenaAsma AyazMeng CuiAsma MaqboolMeng ZhuAsmaa Mohammed HassanMengfei LiAsta JudzentieneMengge DongAsuka IshiharaMengshi LiAthanasia PrintzaMengxi ShenAthanasios KimbarisMeral SertelAthanasios PapadopoulosMercedes MuñozAthanasios PapakyriakouMershen GovenderAtheesha SinghMeta SternišaAthena AndreouMian Abdur Rehman ArifAtiyeh AbdallahMian NavedAtsushi FujimuraMiao-Hsia LinAtsushi SanoMiaojin ZhouAttawit KovitvadhiMiaomiao LiAttila FrigyMicah GrossAudrey GuenicheMichael A. E. RamsayAugusto CerquaMichael AbendAurel MaximMichael Anton BauerAurel Popa-WagnerMichael AppellAurelia Magdalena PisoschiMichael C. GranatoskyAurora Castro-MendezMichael ChristAussie SuzukiMichael ConnollyAustin L. LagrowMichael D. GrayAvinash Chandra PandeyMichael D. SunshineAvinash PremrajMichael GlockerAvneesh GautamMichael J. EbleAxel SteigerMichael L. BernardAyako WashioMichael LakatosAyesha TahirMichael McmurrayAyman ElsamanoudyMichael OrthAyman MostafaMichael PyatibratovAyrat U. ZiganshinMichael RetskyAyse KoseMichael SchirmerAyush DograMichael ScholzAzahara Rodríguez-LunaMichael T. HenzlAzhagiya Singam Ettayapuram RamaprasadMichael TanzerAzis SitanggangMichael TellierAziz BachaMichael ZechB. SpeculandMichail RovatsosBahai JiaoMichał KulusBahareh NowruziMichał SzczyrekBailin ZhaoMichał ZałȩckiBal Krishna ChaubeMichel HananiaBalamurugan ShanmugarajMichel JancloesBalawant KumarMichel MondainBalik DzhambazovMichela Di NottiaBalint GergelyMichela FerrucciBang-Gee HsuMichela GeminianiBanu KaraMichele BoffanoBanzeer Ahsan AbbasiMichele CarboneBaoguo ZhangMichele ColaciBaoshan MaMichele CovelliBappaditya ChandraMichele Di CosolaBaptiste LeroyMichele FioreBarbara GierlikowskaMichele GuidaBarbara JasiewiczMichele MalaguarneraBarbara ŁapińskaMichele MercurioBarbara LipińskaMichele PietragallaBarbara MarengoMichelina IacovinoBarbara ParoliniMickael EssoumaBarbara PawłowskaMickael OhannaBarbara SymanowiczMieke DaneelBaris UzildayMieszko WieckiewiczBarkat KhanMiglė BacevičienėBart De GeestMiguel A. OrtegaBart KahrMiguel A. PeñalvaBart PijlsMiguel Angel Alcántara-OrtigozaBartlomiej BarczynskiMiguel Ángel Mendoza-CatalánBartłomiej ZieniukMiguel Armengot-CarcellerBartosz CzubaMiguel BeltranBartosz ForoncewiczMiguel Castelo-Branco SousaBartosz MałkiewiczMiguel GonzalezBartosz SzmydMiguel Nobre MenezesBaseer U. AhmadMiguel Portillo-EstradaBasem S. ZakariaMiguel Rebollo-HernanzBassam YaghmourMiguel Vicente-ManzanaresBayu Satria WiratamaMiguel WeilBeata Brajer-LuftmannMihaela HedeșiuBeata MessyaszMihaela MureşanBeata Morak-MłodawskaMihai JuncarBeata Mrozikiewicz-RakowskaMihai PopescuBeata Sarecka-HujarMihaiela Cornea-CipciganBeata Świeczko-ŻurekMihail Lucian BirsaBeata ŻołnowskaMihajlo JakovljevicBeatrice GalloMihály JancsóBeatrix DienesMi-Hee KwackBeatriz Gómez-MartínMiho JeongBebiana CondeMiikka-Juhani HonkaBecky HessMika SakamotoBee K. TanMike Yw KwanBehnam Asgari LajayerMikeljon NikolichBehnam VafadariMiklós MézesBekir KabasakalMikołaj DąbrowskiBekir KocazeybekMilan KolářBelaghihalli N. GnaneshMilan SkalickýBelgin SeverMilan SkitekBelkheir HammoutiMilena CavicBen Z. KatzMiles LangeBenjamin E. Nilsson-PayantMilica JankovicBenjamín FloránMilica PopovicBenjamin SilverbergMilijana JanjusevicBenjamin SullivanMilka MaleševićBenjamin ThoreauMilka MilevaBent EhresmannMilorad DragićBernadeta ChyrchelMilorad TesicBernard CanaudMilos LazarevicBernát GáborMilton Ozório MoraesBernd StratmannMilton RoyBernhard EllingerMin XieBert-Ram SahMine KöktürkBertrand TchanaMinerva Codruta BadescuBeshay ZordokyMing YangBharathi DevarajMing-Chih ChiuBhawna DevMingchung ChouBhik KotechaMing-Fen LeeBhola PradhanMingkwan DoilomBiagio BaroneMinglei GuoBiagio MorettiMingna LiuBianca B. KojinMingyi WangBibekananda KarMin-Ho HanBibhu Prasad PandaMinkyung BaekBibhuti B. DasMin-Wook KimBibiane Steinecker-FrohnwieserMiodrag GužvićBijay BeheraMircea TampaBiljana BalenMirela SucheaBiljana M. Todorović-MarkovicMirela TiglisBimal Kumar GhimireMiren AltunaBin GuoMirjam GerwingBindu SubhadraMirna JovanovicBing-Hung ChenMiroslav ChovanecBingqian QuMiroslav P. IlićBinh P. NguyenMiroslav RadenkovicBinlin WuMiroslava NedyalkovaBipasha BoseMiroslava RakocevicBiswajita PradhanMirosław GiurgBiswaranjan MaharanaMirosław TyrkaBjörn KemperMirosława ChwilBjörn RathMiroslawa PuskullogluBlanca R. LopezMiruna NemeczBo WangMirza PojskicBoaz BarakMislav RadicBob LauderMitra AkbariBogdan SoceaMitra SoleimaniBohdan SchneiderMitsuhiko TakahashiBojan ZagrovicMobeen RehmanBoleslaw KarwowskiMoeti Oriel TaioeBolívar Samuel Sosa-MadridMohamad Hesam ShahrajabianBongjoong KimMohamad Koohi-MoghadamBora KaydanMohamed Abdel-AzizBorislav BanjacMohamed Ait-El-MokhtarBoryana Nikolova-MladenovaMohamed AlkafafyBouke P. C. HazenbergMohamed Ashour FikryBowen WangMohamed DkhilBoyan P. ZlatkovMohamed El-MokhtarBo-Young HongMohamed ElmonemBożena Romanowska-DixonMohamed El-SherbinyBożena Szyguła-JurkiewiczMohamed F. M. IbrahimBrach PostonMohamed FawzyBrad BarnettMohamed G. HassanBrandon Lucke-WoldMohamed HashemBrandon Mark GassawayMohamed HassanBranislav OlahMohamed L. AshourBranislav ŠilerMohamed MohanyBranko AleksicMohamed O. RadwanBrega CarlottaMohamed SheteiwyBrent M. ZnoskoMohamed SolimanBrett BlighMohammad Abdul Momin SiddiqueBrian E. StaveleyMohammad AlamBrian GabrielliMohammad Esmaeil BarbatiBrian K. FoutchMohammad HajeerBrianna HarfmannMohammad HossainBrigitte AltmannMohammad Javed AnsariBrigitte KaufmannMohammad MoniruzzamanBrijeshkumar PatelMohammad Nazmul IslamBruno AmatoMohammad QneibiBruno ChrcanovicMohammad Rahimi-GorjiBruno Ferreira VianaMohammad RezaeiBruno FiondaMohammad Robiul HossanBruno Oliveira Da Silva DuranMohammad ShboolBruno PaganoMohammad Tahir SiddiquiBuddolla Anantha LakshmiMohammad TauviqirrahmanBuhari HabibuMohammed AliBulat RamazanovMohammed BuleBurcu Balcı-HaytaMohammed JafarByron Morales-LangeMohammed KoubitiByung Soo KimMohammed Muqtader AhmedC. AlauzetMohammed Oday EzzatCaiyun ZhaoMohammed Zahedul NizamiCălin CăinapMohanraj SadasivamCalin CorciovaMohd AdnanCalogero CasàMohd Adnan KausarCalogero PagliarelloMohd Nazam AnsariCamelia Vidita GurbanMohini GuleriaCanel EkeMohmed Isaqali KarobariCara TimpaniMohsen BakouriCarl BeckerMohsen HesamiCarl Friedrich ClassenMohsen NiazianCarl Johan FürstMohsin JafriCarla CisternasMoises Di SanteCarla Sofia Garcia FernandesMoisés Rubio-OsornioCarla SteccoMojca KržanCarlo AprileMojtaba KeikhaCarlo BizMojtaba KordrostamiCarlo GenoveseMojtaba ZiaeeCarlo José Freire OliveiraMomcilo JankovicCarlo LaiMona K. E. QushawyCarlo Maria AlfieriMoneisha GokhaleCarlo MusioMoni RoyCarlo PerisanoMonica BaroneCarlos AmeroMonica BettaCarlos BicudoMonica CappellettiCarlos Eduardo Da Silva OliveiraMonica HârțaCarlos Eduardo Fonseca-AlvesMonica NavarroCarlos Eduardo FontanaMónica OteroCarlos Fontes RibeiroMonica ValentovicCarlos GarcíaMônica Volino-SouzaCarlos H. HirokiMonika A. SłowińskaCárlos L. Ayán PérezMonika BłaszczyszynCarlos LifschitzMonika CzerwinskaCarlos López-De-CelisMonika KamińskaCarlos O’Connor-ReinaMonika KoziełCarlos OrtizMonika Les̈KiewiczCarlos Rolando Ríos-SoberanisMonika Michalek-ZrabkowskaCarlos RosasMonika Morawska-KochmanCarlotta PipoloMonika TrojanowskaCarmelo MilitelloMonique Van ProoijenCarmelo Ortega RodríguezMonoj Kumar DasCarmen Eugenia SîrbuMoonkyoo KongCarmen Lăcrămioara ZamfirMorag LewisCarmen LageMorena PetriniCarmen Lidia ChitescuMorihito TakitaCarmen Méndez HernándezMoshe SalaiCarmine FinelliMostafa Yuness Abdelfatah MostafaCarmine IzzoMotaz HamedCarmine PiconeMourad A. M. Aboul-SoudCarolina G. PoitevinMrinal BhattacharjeeCarolina OteroMrinmoy GhoshCarolina Susana CerrudoMrutyunjay JenaCaroline Allix-BeguecMubarak Taiwo MustaphaCaroline SubraMubasher HussainCarrubba ElisaMuddasarul HodaCasey WilliamsonMuhamed FočakCassandra FlemingMuhammad AfzaalCassiano Felippe Gonçalves-De-AlbuquerqueMuhammad Ahsan AltafCassone GiuseppeMuhammad Ahsan AsgharCatarina MirandaMuhammad Ajmal BashirCatello ScaricaMuhammad Ali RazaCatharina Sagita MoniagaMuhammad Altaf KhanCatherine GuynetMuhammad Asif NawazCatherine M. MillerMuhammad AzeemCátia DominguesMuhammad HarrisCauê Benito ScarimMuhammad Iftikhar Ul HusnainCaven M. MnisiMuhammad ImranCécile FeuillieMuhammad IrfanCecilia PerinMuhammad Nadeem HassanCecilia XimenezMuhammad NaeemCélio José Castro-JuniorMuhammad NawazCengiz OzalpMuhammad QasimCennet OzayMuhammad Salman FaisalCerasela Elena GîrdMuhammad Siddique AfridiCesar Leobardo Aguirre-MancillaMuhammad WaqasCesar Marcial Escobedo-BonillaMuhammad WaseemCesar Menor-SalvanMuhammed A. AcikgozChai Hong RimMuhammed AtamanalpChaicharn PothiratMuhammed GerçekChaima Alaoui JamaliMuhammed KibriyaChairat NeruntaratMujahid FaridChairmandurai AravindrajaMukesh Kumar SriwastvaChakraborty SohiniMukund V. DeshpandeChalermpong SaenjumMumtaz AnwarChan Soon ParkMurali MamidiChander Shekhar MukhopadhayayMuralidharan ManiChandika GamageMurat Şakir EkşiChandrabose SelvarajMurat ToprakChandrasekhar YadavalliMustafa SevindikChang Eon ParkMustafa ShukryChang LiMustafa SirlakChang Yeon YuMustafa VayvadaChang-Chi LinMuthu ThiruvengadamChanggui LiMuthuramalingam PandiyanChang-Hyun KimMuzammal RehmanChangqi LiuMya Myat Ngwe TunChanna Maria AloisiMykola MamenkoChanon NgamsombatMyriam CayreChanpreet SinghMyriam RichaudChantelle AhlenstielN. H. SchumakerChao HeN. Jeelan BashaChao XiaNabeel AhmedChao ZhangNabil ElsheeryChaoqi ChenNadav MichaanChaoqun HuangNadica Maltar-StrmečkiChaowalit YuajitNadin ElsayedCharalambos FotakisNadir Nadir ZamanCharalampos ThomasNafisa H. BalasinorCharat ThongprayoonNaga Raju MaddelaCharikleia KelaidiNaga Swetha SamjiCharis NtakoliaNagentrau MuniandyCharmaine Van EedenNahid AboutalebChellappagounder ThangavelNa-Hyung KimChen Liang TsaiNalan Oya San KeskinChendong HanNan WuCheng Hong TsaiNancy CasanovaCheng WangNancy LinCheng XueNancy S. YounisChenggen ChuNan-Jay SuCheng-Hsien LiuNannan LaiChenjian GuNaoki AkamatsuChenjuan GuNaoki NagataniChenlu GaoNaoki OharaChenyang CaiNaoki WatanabeCheryl R. MccrearyNaoki YamamotoChetan PaliwalNaoko KatsuradaChetna SoniNaomi KakoschkeChhagan BihariNarayan BhusalChia-Chi YangNarayanankutty ArunaksharanChia-Chu ChangNarendiran RajasekaranChianju JongNaresh KumarChiara AmitranoNaresh Kumar MeenaChiara BiselliNarongchai AutsavaprompornChiara MoltrasioNashwa HagagyChiara PanicucciNasim NaderiChieko AzumaNatalia Díaz-ValdiviaChien-Tai HongNatalia Ivanovna AgalakovaChien-Yu LinNatalia KazninaChih-Chin YangNatalia KrawczynskaChih-Hsiung HsuNatalia KurhalukChih-Li LinNatalia MotasChin-Chean WongNatalia PerunovaChing-Chung LinNatalia SimionescuChing-Jin ChangNatalia V. BelosludtsevaChinmay V. TikheNatalia Viktorovna NizyaevaChinmaya MahapatraNatalie H. MatthewsChi-Rei WuNataliia V. AnnenkovaChi-Young WangNatalya GlushkovaCh'Ng Huck YwihNataša Marčun VardaChong Chin HeoNatsuo UedaChong-Tai KimNattawut BoonyuenChoo Hock TanNavapon TechakriengkraiChris Hl LimNaveed IqbalChrissa SiokaNaveen DixitChristen L. EbensNavid AbedpoorChristian BarbatoNavin Kumar DevarajChristian FercherNavved AhmedChristian GrebmerNawroz TahirChristian HappelNazanin Ebrahimi-AdibChristian JungnickelNazanin-Zahra Shafiei-JandaghiChristian LehmannNazarii KobyliakChristian MayerNderitu PaulChristian R. Encina-ZeladaNeal BurtonChristian SteuerNebojsa IlicChristian-H HeegerNebojša JasnićChristina MejersjöNeda AničićChristine SilvainNeda BaghbanChristof RöösliNeda HakimihaChristof RottenburgerNeda MohamadiChristoph BorzikowskyNeda SladeChristoph CastellaniNeha GuptaChristoph FaschingerNeha KoonjooChristoph KlenkNeil McgregorChristoph ReinhardtNejc PikoChristopher A. MakaroffNejc UmekChristopher H. HouseNejka PotočnikChristopher J. YoungNektarios KalyvasChristopher M. WilliamNemany Abdelhamid Nemany HanafyChristopher Munoz-BendixNémeth BalázsChristos AnagnostouNenad JoksimovićChristos FeloukidisNenad PandakChristos K. YiannakopoulosNermeen YosriChristos RumbosNessma Ahmed El-ZawawyChrysoula NtislidouNeusa Yuriko Sakai ValenteChuancheng WuNeval OzkayaChuang-Wei WangNeven MatočecChuanming XuNeven PapicChul-Su YangNguyen Minh DucChun ZhangNguyen Quoc KhuongChunchia ChengNibedita PatelChung-Hsin WuNicola DecaroChun-Ren WangNicola Hamilton-WhitakerChushuang ChenNicola IelapiCinzia FerrariNicola MarottaCiprian RoiNicola MarranoCiro EspositoNicola MondanelliCiro ImbimboNicola PuglieseClair VandersteenNicolae BacalbașaClaire LeconteNicolae GicaClaire ProvostNicolas R. De SouzaClaire Stines‐ChaumeilNicolas SayeghClara AzzamNicoleta AntonClara IannuzziNicoleta RaduClaudia BonfioNicolette HoureldClaudia ContiniNidal JaradatClaudia DesiderioNiels Grote BeverborgCláudia FarinhaNiels Thue OlsenClaudia KusmicNighat PerveenClaudia LermaNihal ÇetinClaudia MehedintuNihat AkbulutCláudia RochaNikhlesh SinghClaudia Z. HanNikita KlugeClaudio Acuña-CastilloNikola Hadzi-PetrushevClaudio CappelliNikola SekulovskiClaudio De LazzariNikola StojanovicClaudio FerranteNikola UnkovićClémence BaudinNikolai RamadanovClemence RougeauxNikolaos A. ChrysanthakopoulosClemens FeistritzerNikolaos E. TzanakisCleverton De AndradeNikolaos KatzilakisClifford F. BrunkNikolaos NikoloudakisColagiero MariantoniettaNikolaos RemmasColombina VincenziNikolaos SiderisColtri Priscila P.Nikolaos StalikasConcetta Di NoraNikolaos VlahosConcettina La MottaNikolay A. PyataevCong XieNikolay NityagovskyConstantina PolitisNikoleta PrintzaCorina VasileNikos KrigasCornelia De MoorNilakshi BaruaCornelia MirceaNilanjan ChakrabortyCorrado CiattiNilesh ChoudharyCorrado MagnaniNilesh J. PatelCorrado PelaiaNilesh WashnikCosimo GallettiNileshi SarafCriscuolo ElenaNils BömerCrismita DmelloNima SanadgolCristi TartaNina L. PetrovaCristian DeanaNipun Lakshitha De SilvaCristian FrancavillaNirajan ShresthaCristian PersuNiroj Kumar SahooCristian StătescuNishant KulkarniCristian TrâmbițașNitikaCristiana Iulia DumitrescuNitin TelangCristiana SoldaniNitinkumar BorkarCristiane AguiarNizar TliliCristiane Angélica OttoniNobumichi KobayashiCristiane Damas GilNoelia González-GálvezCristiano J. De AndradeNoemí Cárdenas-RodríguezCristina AchimNoha H. HabashyCristina Alvarez-PeregrinaNooshin SohaniCristina Anna Maria Lo IaconoNoradin GhadimiCristina CantaruttiNorbert KissCristina CapatinaNoriaki MaedaCristina CasalouNorifumi YamamotoCristina Castro-AlonsoNorihiko NaritaCristina GluhovschiNorimichi KoitabashiCristina GuarducciNosir ShukurovCristina Iulia MitranNouran Yousef SalahCristina LullNuning NurainiCristina MihaliNuray AcarCristina OlivaroNurdjannah Jane NiodeCristina PopNurgul YurtsevenCristina SegoviaNuria Conde-PueyoCristina SoaresNuria P. Torres-AguilaCristina Souza Freire-NordiNurit ShtoberCristina TruzziNurlaila IsmailCristoforo PomaraOana AlmasanCsaba VagvolgyiOana CioancaCuauhtémoc Sáenz-RomeroOana-Maria BolduraCuk-Seong KimOctavian Tudorel OlaruCynthia BlantonOfer BeeriCyril FersingOkezie EmmanuelCyrus MotamedOlaf GefellerD. Mohd. Azam AnsariOleg I. BaryginDa SunOleg T. DevinyakDaehoon LeeOleg V. GradovDae-Wui YoonOleksii SkorokhodDag NymanOlga A. KoksharovaDagmar PavlůOlga A. SinitsynaDaiji EndohOlga A. SnytnikovaDaiji NagayamaOl'Ga A. TarasovaDaisuke KobayashiOlga AnatskayaDaisuke TodakaOlga BrovkinaDaisuke TsurutaOlga Czerwińska-LedwigDalia MedhatOlga KopachDalila ScaturroOlga TsivilevaDallas SeitzOlga Warszawik-HendzelDamien VitielloOlivera Mitrović AjtićDamir FabijanićOlivier BeufDamir SuljevićOm Prakash ChoudharyDan C. VodnarOmair MohiuddinDan ChenOmar AlzubiDana CairnsOmar Hussein SalmanDana GhigaOmar JanehDan-Bogdan NavolanÖmer EkmekcioğluDandan SunOmid MirmosayyebDangdang LiOmid SafariDanh C. VuOmneya AttallahDaniel A. MedinaOndřej MalýDaniel Almeida MarinhoOndřej PlíhalDaniel ArcanjoOng Ardvin Kester SDaniel Ardisson-AraújoOreste MarroneDaniel Cerqueda-GarcíaOrietta PansarasaDaniel Cervantes-GarcíaOrnella CominettiDaniel CookOscar CampuzanoDaniel ElbirtOscar CruzDaniel F. SalamoneOscar Martínez-PérezDaniel FinkeOscar Mauricio Martínez AvilaDaniel Hernandez-PatlanOsman TaylanDaniel KretzschmarOsvaldo MazzaDaniel L. PiskorzOttavio TomasiDaniel LemireOttmar HerchenröderDaniel López MaloOumei ChengDaniel López-LópezOussama SaidiDániel NemesOvidiu PopDaniel OhOxana BelovaDaniel P. CardinaliOyuna KozhevnikovaDaniel P. PotaczekOzge SahinDaniel P. ZalewskiOzgur Koray SahingozDaniel PatschanOzren PolašekDaniel RamsköldÖzüm Yuksel TunçyürekDaniel RemiasPablo G. Del RíoDaniel Robert SchlattererPablo RuizDaniel RodriguezPablo Torres-VergaraDaniel Sánchez-CanoPa-Chun WangDaniel SedorkoPajaree TotakulDaniel ŚlęzakPakeerathan KandiahDaniel TurudićPalma ScolieriDaniel Vasile BalabanPambayan Ulagan MahalingamDániel VéghPamela BurgerDaniel Xin ZhangPamela EhrenfeldDaniela AmzarPanagiotis SimitzisDaniela Carmen AbabeiPanayiotis LoukopoulosDaniela Di VenerePankaj AhluwaliaDaniela HaluzaPankaj KumarDaniela Joyce Trujillo SilvaPantelis KourosDaniela LeccaPaola De LucaDaniela Ledić DrvarPaola FerrariDaniela MarzioniPaola LenziDaniela Opris-BelinskiPaola PiomboniDaniela QuaglinoPaola QuaresimaDaniele Filippo CondorelliPaolo Albino FerrariDaniele GarcovichPaolo ArosioDaniele GhezziPaolo BennaDaniele Giovanni GhiglioniPaolo Boscolo-RizzoDaniele MasaronePaolo CastiglioniDaniel-Genel SurPaolo DolzaniDanielle B. L. OliveiraPaolo FagoneDanielle BorgPaolo FiorinaDanielle FribergPaolo MarianiDanielle K. OfferdahlPaolo MereuDaniil OlennikovPaolo NardiDaniil P. AksenovParamjit TappiaDaniil SergkelidisParaskevi TrivilouDanijela ArsenovParasuraman PavadaiDanijela TasicParham Jabbarzadeh KaboliDanila BobkovParham Mohsenzadeh KebriaDanilo Sipoli SanchesParimalan RanganDanko MiloševićParisa GazeraniDaoping HeParisa ZiaratiDarek GoreckiParisi AttilioDaria BylievaParmjit S. JatDarinka AckovaParveen Fatemeh RupaniDario AchaPascal Alexandre ThomasDario CalafiorePascal ProbstDario Di NardoPasquale CaldarolaDario MizrachiPasquale MoneDario SiniscalcoPasquale PiombinoDariusz KucharczykPatricia CantoDariusz KulusPatricia Doyle-BakerDariusz PiesikPatrícia NicolucciDarko AntičevićPatricio ArtigasDarren TanPatricio MuñozDaryl BowmanPatrick D. MathewsDave BoucherPatrick G. ArndtDavid A. BaumPatrick H. MassonDavid A. GerberPatrick O'DonoghueDavid CluckPatrick SchussDavid DolivoPatrik Ferreira VianaDavid GrynspanPatrizia AmadioDavid J. MaddenPatrizia PorcuDavid LibichPatrizia RussoDavid Li-KroegerPatrizia ZaramellaDavid M. DiamondPatrycja KleczkowskaDavid Manzano-SánchezPaul A. SievingDavid MartinezPaul DahlinDavid P. RemetaPaul F. WilliamsDavid RayPaul G. HiggsDavid Sancho-CantúsPaul M. BartleyDavid SergeevichevPaul Mark Baco MedinaDavid SteffenPaul OtyamaDavid VětvičkaPaul SchoenhagenDavid W. PowellPaul WiseDavid ZemanPaul Zavala-RiveraDavide BiancoPaula AntonoaeaDavide BizzocaPaula BoaventuraDavide BochicchioPaula Daza-NavarroDavide CostaPaula Manuela Mendes Moleirinho‐AlvesDavide Giuseppe RibaldonePaul-Mihai BoarescuDavide LeardiniPaulo A. GameiroDavide MedicaPaulo Eduardo TeodoroDavorin SefPaulo Magno Martins DouradoDawid PrzystupskiPaulo PalmaDe Gaulle I. ChigbuPaulo R. FernandesDean TangPaulo SchwingelDebadatta SethiPaulo ZainiDebashis DuttaPavan Kumar DhanyamrajuDebasis MitraPavel A. DmitrievDebasish BasakPavel KerchevDebasmita MukhopadhyayPavel KroupinDebojyoti MoulickPavel PashkovskiyDébora FerreiraPavel StudenýDebora NapoliPavel TarazovDeborah SchechtmanPavel VítámvásDeeksha BaliPavol PobehaDeepak Kumar KhajuriaPawan Kumar RaghavDeependra Kumar SinghPaweł ChmuraDeepika AwasthiPaweł GaćDeepika SinglaPaweł KordowitzkiDeguan LvPaweł KrukowDehong YanPaweł OlczykDemetrio MilardiPaweł P. PomastowskiDenis A. BabkovPayal GangulyDenis HarkinPedro Cerezal-MezquitaDenis KolbasovPedro FonsecaDenisa HazballaPedro Martín-AcostaDenise HillingPedro Mendoza De GivesDennis NürnbergPedro MorouçoDeogratius LuyimaPedro PellicerDésirée ForstnerPedro PiedrasDevarajan BharanidharanPeihua MaDeven PatelPeik Lin TeohDevendra MaheshwariPei-Ling HsiehDhananjay YadavPei-Luen LuDharmendra KumarPejman RohaniDhriti SinhaPeng ChenDhruba PathakPeng LiDi WuPengbo ZhangDiaa MoneimPengfei JiaDiana Andreia Tavares PintoPengpeng LiuDiana AntalPensieri SaraDiana HendrickxPeramaiyan RajendranDiana López-AlvarezPernilla StenströmDianne GreenfieldPerry B. HackettDibya PandaPetar OzretićDidik Setyo HeriyantoPete N. MonkDiego CotellaPeter BoltucDiego SaukaPéter CsécseiDieter BraunPeter FranklinDietmar KültzPeter G. RomaDileep KumarPeter HauserDillirani NagarajanPeter PetzelbauerDimas De Barros SantiagoPeter Ping LinDimitri PoddighePeter VitielloDimitrios I. RaptisPeter WaldeDimitrios KanelisPetr MlejnekDimitrios KotsirisPetr PyszkoDimitrios T. PapadimitriouPetra ImhofDimitris EmfietzoglouPetra Peharec ŠtefanićDina YarullinaPetra PetranovicDinesh Kumar VermaPetro E. PetridesDingcheng GaoPetros PapalexisDiogo Goulart CorrêaPhatu MashelaDionysios VorosPhilip SarajlicDiotallevi MarinaPhilipp E. ChetverikovDipak Kumar SahooPhilipp J. PoxleitnerDipali SrivastavaPhilipp MinzlaffDirk Schulze-MakuchPhilipp RauschDivya GopinathPhilippe D'AbadieDmitri ShchekochikhinPhilippe De DeurwaerdereDmitriy A. ParkhomenkoPhilippe NgheDmitriy RudoyPhillip DettleffDmitriy V. MazurovPia Lopez-JornetDmitry A. KnorrePia R. NielsenDmitry GarbuzenkoPiera Anna MartinoDmitry KarpovPiero Antonio ZeccaDmitry SitnikovPiero PaladiniDmitry SychevPierre TennstedtDo Nascimento Carla FerreiraPietro BertoglioDoerte WichmannPietro CacialliDo-Hee KimPietro FransveaDolores CidPietro ManiscalcoDolores Remedios SerranoPietro ScicchitanoDomagoj VidosavljevicPietro SpennatoDomenico RongaPing SuDominik DłuskiPing ZhuDominik SaulPingxian ZhangDominique De ClercqPingyuan WangDomitilla MandatoriPiotr AugustyniakDomiziano TarantinoPiotr BrągoszewskiDonatella Degl'InnocentiPiotr ChmielarzDong Keon YonPiotr DuchnowskiDong ZhuPiotr GawdaDong-Hyun KimPiotr KanclerzDongmei LiPiotr KapustaDongxian GuanPiotr KuropkaDora Luz OjedaPiotr MorasiewiczDora TangPiotr Samel-KowalikDoris RusicPiotr SzwedaDorival Mendes Rodrigues JuniorPiotr SzymczykDorota NowakPiotr WlaźDorota Wilk-KołodziejczykPiotr WychowańskiDorota WojtysiakPires IvanDorothea TsekouraPiyush BaindaraDoungporn AmornlerdpisonPlamen KatsarovDov BorovskyPonnuraj Nagendra PrabhuDragan HrnčićPooja SinghDragana BjelicPoonam C. SinghDragana D. PredojevićPorin PerićDragana LazarevićPoulami DattaDragana M. StanojevicPrabakaran NagarajanDragana ProticPrabhakar BhandariDragica SelakovicPrabhugouda SiriyappagouderDragos Catalin JianuPrabu Kumar SeetharamanDragos CozmaPradeep RaiDragos Paul MihaiPrahlad HoDriss OusaaidPrajish IyerDrozdstoy StoyanovPrakash Chand NegiDulce Maria Palmerin-CarreñoPrakash KarmakarDumitru CiolacPrasanna Kumar SanthekadurDuncan ToplissPrasanna RamaniDunja SamecPrasannavenkatesh DuraiDuo ZhangPrasanthi KogantiDurga Kalyani PaturiPrashanth SuravajhalaDustin OranchukPrathyusha BagamDyana OdehPratibha KumariDylan Jack RichardsPraveen C. VermaDylan PeukertPrawej AnsariE. Begoña Garcia-NavarroPrawin KumarEberhard HildtPreetha SharanEbrahim Abbasi OshaghiPrem KumarEbrahim KouhsariPrem Raj MeenaEbrahim ShokoohiPrem SagarEbubekir YükselPrema Latha MallipeddiEd Van BavelPrescott DeiningerEdgar Julian Paredes-GameroPrinpida SonthiphandEdgard DelvinPriscila Nasser De Carvalho KrollEdmund KoziełPritam SadhukhanEdmundo Bonilla GonzálezPritesh DesaiEdmunds ReineksPrithwi GhoshEdoardo ConticiniPriyank Hanuman MhatreEdoardo Gioele SpinelliPrzemko KwintaEdson TalaminiPrzemyslaw ChmielewskiEduard MuraniPrzemyslaw PlonkaEduardo Gomez-BañuelosPugazhenthan ThangarajuEduardo José Veras LourençoPukar KhanalEduardo Pandolfi PassosPuneet DhamijaEduardo Valencia CanteroPuneetpal SinghEdward KoifmanPurvi ChawlaEdyta PasickaQamar Abbas SyedEfstratios StratikosQasem Al-TashiEhab AbdelhieeQi JinEhsan DowlatiQi WangEiichi KumamotoQiang LiuEija NissiläQibin GengEiji NagayasuQichang MeiEka PrasedyaQin FuEkaterina A. LitvinovaQin WuEkaterina BaranovaQing WangEkaterina OrlovaQinghui WangEkaterina RudnitskayaQingjun PanEkaterina SukhovaQinglan DingEkaterina V. MedvedevaQing-Quan ZuEkaterina V. ZubarevaQingsen ShangEkaterina YurchenkoQingyu ZhouEladl EltanahyQixiang LinElaine DunlopQuang Huy NguyenEl-Anwar Mohammad WaheedQuanwei ZhangEldad DannQudrat UllahEldrin D. ArguellesQun LuoEleftherios AgorogiannisR. KocyłowskiElena AnghileriR. Martin VabulasElena ApostolovaRabia Sannam KhanElena AvdeevaRachna KapoorElena Balestreire HawrylukRade M. BabićElena BernadRadha GopalaswamyElena Bueno-GraciaRadoslava KralevaElena CaporaliRadosław GocołElena CicheroRadosław KujawskiElena D. BazhanovaRadu ChiceaElena KashubaRadu RacovitaElena Maria VaroniRadu-Stefan MiftodeElena SavvateevaRaees KhanElena SilvestriRafael Block SamulewskiElena TarcaRafael MendesElena V. ProskurninaRafael Scaf De MolonElena VianelloRafał Białynicki-BirulaElena Victorovna DeinekoRafał JanuszekElena ZhurikovaRafał MichalskiEleni KakouriRafal OgorekEleonora GaetaniRafał OgórekEleonora MacediRaffaele CapassoEleonora Maria TreccaRaffaele SerraEleonora OrtuRaghu P. KataruEleonóra SpekkerRahul DattaElga BandeiraRai Campos SilvaElham HosseiniRaj Kishori LalElham KezarooniRaj KumarElham MasoumiRaj Kumar MongreElham PishavarRaj PaudelEli FeiringRaja Gopal Reddy MooliElia GattoRajalakshmi SubramaniyamEliel Ruíz-MayRajan KashyapElif KaraaslanRajeev K. SinhaElisa BannoneRajeev RathiElisa BelluzziRajeev SinglaElisa CairrãoRajendra AcharyaElisa ReitanoRajendran VelmuruganElisa SalaRajesh K. TripathyElisa ScarsellaRajesh KasamElisa ScarselliRajesh Kumar TripathyElisabet A. ForsumRajesh ParsanathanElisabet Navarro-TapiaRajesh YetirajamElisabeta AntonescuRajkumar P. ThummerElisabete SilvaRajkumar SahaniElisabeth PinartRajmohan DharmarajElisabetta AffabrisRajneesh JaswalElisabetta MurruRajnibhas Sukeaw SamakradhamrongthaiElisabetta PolizziRajnikant DixitEliška SovováRakhi IssraniElizabeth DavisRalf DuerrElizabeth HicksRaluca DumacheElizabeth Ortiz-SánchezRamachandran SivaramakrishnanElizabeth PearceRamalingam BethunaickanEl-Kazafy Abdou TahaRamanna Hunashikatti LaxmanElod NagyRamcharan Singh AngomElon H. C. Van DijkRamendra Pati PandeyElpida-Niki Emmanouil-NikoloussiRamesh KumarElsayed MansourRamesh MukkamalaEly Oliveira-GarciaRamesh PudakeElżbieta Łodyga-ChruścińskaRamesha PapannaElżbieta Łopieńska-BiernatRamgopal KashyapElżbieta PatkowskaRamin JaberiElzbieta SalinskaRamin SalimnegadElzbieta Studzinska-SrokaRamnik V. PatelEman E. MahmoudRamón C. HermidaEman MehannaRamón González-CamarenaEmanuela SalviatiRamona DanacEmanuele RinninellaRanajay SahaEmerson Alves Da SilvaRangaswamy MadugunduEmil N. SobolRaphael Martins De AbreuEmilia IannilliRaquel De Cássia Dos SantosEmilie WidemannRaquel Moreno LoshuertosEmilio VanoliRareş Mircea BîrluţiuEmily E. MatulaRasheedaunnisa BegumEmina Sudar-MilovanovicRashid MirEmine IkbalRasmus Hvidbjerg GantzelEmira NoumiRasoul MirzaeiEmmanouil GiorgakisRatchadawan AukkanimartEmmanouil KapetanakisRaul MartinEmmanuel BourdonRaúl Pérez LlanesEmmanuel J. FavaloroRavesh SinghEmmanuel Navarro-FloresRavi Chakra TuragaEmmanuel NonyRavi P. SahuEmmanuelle Gilot-FromontRavi PandiselvamEmrah YatkinRavi Pratap SinghEmre BilginRavi Shankar ReddyEmyr PeñaRavinder KumarEnbo MaRay MannehEngy ElekhnawyRay W. BasrowiEnri NakayamaRazvan SocolovEnrica AlicandriRăzvan-Ionuț PopescuEnrico BrunettiRebeca Betancourt-GalindoEnrico ContiRebeca Boltes CecattoEnrico OrsiRebeca González-FernándezEnrico RuizRebeca LorcaEnrico Vito PerrinoRebeca Romo-VázquezEnrique JaimovichRebecca L. MickolEnrique Méndez BolainaReda DjidjikEntesar DalahRedha TaiarEnver Cagri IzguRedi RahmaniEnza SperanzaRegina Fölster-HolstEpaminondas ZakynthinosRegina Guenka Palma-DibbEphrem EngidaworkReginaldo Dos Santos PedrosoEraldo OcchettaRegragui TakiEraldo Sanna PassinoReham HamidaErcan AvşarReid BishopErgun KayaRekha BhartiEric De Camargo SmidtRekha WarrierEric De GrootRemigiusz PaniczEric Kwesi NarteyRenan Paulo MartinEric RytkinRenata Heisler NevesErica ScottRenata Puppin ZandonadiErick Sierra-CamposRenata Tobiasz-SalachErik BehringerRenato B. PereiraErika Liktor-BusaRenato De FilippisErika López-ArribillagaRenato GalzioErika MandaràRenato GiacominiErin H. SeeleyRené BuchetErnesto Moya-AlborRene HuberErnst-Detlef SchulzeRenée Maria BorgesErshuai ZhangReni KalfinEsam YahyaRenlei JiEslam El-SawyRenuka JayatissaEsmaeil Doustkhah HeraghRen-Wang JiangEssa M. SaiedRiadh BadraouiEssam Ahmed Mohammed Al MoraissiRicardo AmilsEssam Al-MoraissiRicardo AraujoEsteban ManriqueRicardo EscalanteEsther F. Vicente-RabanedaRicardo GunskiEsti Handayani HardiRicardo PitaEszter NemeskériRicardo Reyes-DíazEtefa Guyassa DinssaRicardo Ruiz-VillaverdeEto Silas FernandesRicardo Sánchez De MadariagaEtsuo NikiRiccardo GobbiEugene KopantzevRiccardo MastroianniEugene MamontovRiccardo MuscarielloEugenia VlachouRiccardo PozzanEugenio MartelliRicha SalwanEugenio RastelliRichard A. AmosEuishin Edmund KimRichard BodnarEun Ji LeeRichard EgelEunice CarrilhoRichard FunkEurico LimaRichard GordonEva BagyinszkyRichard J. StarkEva FeketeovaRichard M. MariitaEva TvrdaRichard M. NilesEvangelia AntoniouRichard SheardyEvangelia BountouviRichard WagnerEvangelia ChavdoulaRichard WebsterEvangelia GoliaRigers BakiuEvangelia KoukakiRiham M. FliefelEvangelos KoustasRimana Islam PapryEvelina Maria GosavRini AriantiEverlyne M’Mbone MulekeRino BelloccoEverton C. CazzoRisely Ferraz AlmeidaEvgen MultiaRishabh DevEvgenii GusevRishi SharmaEvgeniya BurkovaRita Catalina Aquino CaregnatoEvgeny ZatulovskiyRita PavasiniEwa BrzozowskaRita SerranoEwa DługoszRitesh RamdhaniEwa FiedorowiczRiwanti EstiasariEwa Gibuła-TarłowskaRiyaaz Uddien ShaikEwa KublikRizwan QureshiEwa M. UrbańskaRizwan WahabEwa MisterskaRobert BiczakEwa SawickaRobert CesnjevarEwa TomaszewskaRobert E. SmithEwa Toporowska-KowałskaRobert KarpińskiEwald LindnerRobert M. HermannEwelina DondajewskaRobert M. SiegelEwelina HallmannRobert NaeijeEziuche Amadike UgboguRobert NordstromFabian BartschRobert OssendorffFabián Martínez-HernándezRoberta AdorniFabian TomschiRoberta IbbaFabio BottariRoberta ModicaFabio CampodonicoRoberta MoschiniFabio FerlaRoberta ZupoFabio Laurindo Da SilvaRobertas DamaseviciusFabio SantacaterinaRoberto CasconeFabio ZainaRoberto ChiarelliFabio ZugniRoberto CiampagliaFabrice CompainRoberto ColasantiFabrice DarcheRoberto LatiniFabrice LejeuneRoberto Martínez ÁlvarezFabrice LucienRoberto MinieroFabrizio MartoraRoberto VernaFabrizio ParenteRobson De FariasFahad NasirRóbson TeixeiraFahd RasulRobson X. FariaFahri OvaliRocío Ruiz LazaFaisal AlzahraniRocío TalaverónFakhredin SabaRoderjan João GabrielFalk GühneRodica BălașaFan LinRodica Maricela AnghelFang JunRodney A. MclarenFangjiang ZengRodrigo A. LoiolaFangzhu ZhaoRodrigo Coutinho De AlmeidaFani LadomenouRodrigo ValenzuelaFanpei Gloria YangRoel Van OvermeireFa-Po ChungRogelio González-GonzálezFarida BenhadouRogelio Gónzalez-GónzalezFarideh RaziRoger E. KelleyFarzad KermaniRoger JonesFarzad KianersiRogério Leone BuchaimFarzaneh KordbachehRohollah NasiriFasih Ullah HaiderRoland Van De TillarFatehi FoadRoland WinterFatemeh MirzaeiRomain GuilbaudFatemeh SaidniaRoman LesykFatemeh YazdianRoman NowickiFátima AmaroRoman PiotrowskiFatima El KhalloufiRomana ČeovićFátima Ribeiro-DiasRomeo RomagnoliFatma Nese SahinRomeo Theodor CristinaFazlullah KhanRomeu Cardoso GuimarãesFederica BarbagalloRomualdo Del BuonoFederica FogacciRonald B. BrownFederico BiglioliRonit SionovFederico Manuel GiorgiRoozbeh RajabiFederico RossiRosa MancinelliFederico TessadoriRosa VallettaFei PengRosalba ParentiFei QiRosalia DettoriFei WangRosalva Mora-EscobedoFelice CrocettoRosangela Zacarias MachadoFelipe ParedesRosanna Del GaudioFelix BraunRosario LicitraFelix ReichertRoumita MoulickFelix RommelRowena B. CarpioFelix SchmittRoxana LucaciuFelix VauxRoy L. J. Van WanrooijFengchao LangRubina JibranFengjie SunRuchi AgrawalFerdinando AgrestaRudá Amorim LucenaFerdinando SartucciRudolf KorinthenbergFerenc OroszRudolf LikarFernanda Barros-AragãoRuei-Nian LiFernanda M. SilvaRui HaddadFernando AlmeidaRui MinFernando AriasRui YanFernando Augusto Ugusto Mardiros HerbellaRui YuFernando Capela E. SilvaRuilong ShengFernando FerreroRumesh RanjanFernando Lázaro-PeronaRumiana BakalovaFernando López-CamposRundk HwaizFernando M. RohanRuoxiu XiaoFerran Fece De La CruzRupendra ShresthaFestus Adeyemi AdejoroRupesh DeshmukhFiammetta PiersigilliRupesh Kumar ChikaraFilipe Carvalho De VictoriaRupesh Kumar SinghFilippo MiglioriniRusan CatarFilippo TorielloRustem IlyasovFilippos TriposkiadisRyan LimbockerFiorenzo LupiRyan S. CostaFirdaus HayatiRyo MizuuchiFlávia Aparecida GraçaRyoma MichishitaFlavia DematheisRyousuke SatouFlavia PauriRyszard KierzekFlavio CapraroRyszard SlezakFlavio LicciulliRyuichi OhtaFlor H. PujolRyunosuke HakutaFlora N. BalievaS. H. ShinFlorian GembardtS. Karthick Raja NamasivayamFlorian GesslerS. M. Shakeel IqubalFlorian ReckerS. RamkumarFlorin GorunSaad FaroukFlorin OanceaSaad H. D. MasryFlorin OnisorSaad Ihsan ButtFong-In ChouSaad Naser Al-KahtaniFonseca CassianeSaad TayyabForough JahandidehSaba ShahinFortunato IacovelliSabata MartinoFotis BaltoumasSaber SaedmocheshiFouad BenhibaSabina Antonela AntoniuFoziyah ZakirSabina GaliniakFrancesca AmadoriSabyasachi ChatterjeeFrancesca ArfusoSaccoccia FulvioFrancesca CasciaroSachiko KoyamaFrancesca FerraraSachit AnandFrancesca GranataSadaf JahanFrancesca PrignanoSadanand PandeyFrancesca ValleseSaddam SaqibFrancesco BiancoSadiq HussainFrancesco BorgiaSaed MoradiFrancesco CacciolaSaeed AbroFrancesco Carlo MorabitoSafa ShehabFrancesco CiodaroSagarika MishraFrancesco ClapsSagnik SenFrancesco CucciaSahar A. YoussefFrancesco Di GennaroSai Reddy DodaFrancesco GallettiSaid I. BehiryFrancesco GaziaSaif UllahFrancesco MannelliSairam KrishnamurthyFrancesco MassariSajad AhmadianFrancesco PepeSajad BorzoueisilehFrancesco Pinto BoenoSajad KarampourFrancesco SalzanoSajjad ShiraziFrancesco SartiniSakeenabi BashaFrancesco Saverio LudovichettiSakue MasudaFrancesco Saverio Robustelli Della CunaSalem Youssef MohamedFrancesco ScavelloSalleh AnnasFrancesco SmedileSalman Afroze Azmi MohammedFrancieli RohdenSalvador C. HerreraFrancine Amaral PiubeliSalvatore CambriaFrancine S. KatzSalvatore IaconoFrancisca RodriguesSalvatore ScaccoFrancisco Alejandro SanchezSaman FatemiFrancisco Do ValeSamanta RaboniFrancisco EpeldeSamantha MaurottiFrancisco EspinosaSamar TharwatFrancisco Fabio Oliveira De SousaSamasca GabrielFrancisco Guede-RojasSameer Ahmad GuruFrancisco Guillen-GrimaSameer ChavanFrancisco Javier Alves VasSameh AttiaFrancisco José González-MineroSami Abou FayssalFrancisco José HidalgoSami AkbulutFrancisco LeonSamira TarashiFrancisco MesaSamo FokterFrancisco Navarro-TriviñoSampath Dakshina Murthy AchantaFrancisco Sandro Menezes-RodriguesSamrad MehrabiFranco BassettoSamrita DograFranco CervellatiSamuel Fernández CarneroFranco FulcinitiSamuel PushparajFranco MutinelliSamuel X. ShiFranco PallaSamuele CerutiFrançois BoyerSamuele De BartoloFrançois ChevalierSana AshrafFrank LangerSanaa Mahmoud Metwally ShanabFrank Mayta-TovalinoSanbo QinFrantzeska FrantzeskakiSandeep AmetaFranz LahnsteinerSandeep JagtapFranz ZehentmayrSandeep MalampatiFranziska DenglerSandeep MalyanFrederic JungbauerSandeep SharmaFrederic TortSandeep VijayanFrederick H. SilverSander LefereFredrik WärnbergSandesh PanthiFu Wa LeeSandip N. ChavanFuhai LiSandip S. ShindeFu-Jen ChengSandor BenyheFukun GuiSandor I. BernadFuli LiSandra BerndtFumihiro OgawaSandra BischofG. A. BlaiseSandra Cabo VerdeG. Eric PlumSandra CastilloG. N. VeremeichikSandra Kalil BussadoriG. Raj MurugesanSandra Rajme-LopezG. S. PushkarevSandra SkujaG. VijayaraghavanSandra ZecchiGabriel ArmenceaSang Moo LimGabriel Del RioSang-Han LeeGabriel González-ValeroSanja HromišGabriel MurariuSanjay KalraGabriela AniteiSanjay Kumar GuptaGabriela Castañeda‐CorralSankar ChatterjeeGabriela PetrofSanower HossainGabriela-Dumitrita StanciuSanqiang GongGabriele CentiniSantanu GhoshGabriele FuchsSantiago J. BallazGabriele MeroniSantosh UpadhyayGabrielle DrevetSaowarath JantaroGad DeganiSapna SharmaGaetano SantulliSaqib BashirGagan D. GuptaSara BernardiGaia GoteriSara Casado ZapicoGaku TokudaSara DominguesGalina Grigorievna BorisovaSara MangiaterraGalina NikolovaSara PahlavanGalit AlmozninoSara Remuzgo-MartínezGanesh C. NikaljeSara RicciardiGanesh Dattatraya SarataleSarah E. JacksonGanesh IngavleSarah El-NakeepGang ChenSarah K. LucasGang KouSarah LamereGang PengSarah Louise Haywood-SmallGangarapu KiranSarath VijayakumarGary Garcia-MolinaSaravanan MuthupandianGaspare PalaiaSareesh Naduvil NarayananGassan MoadySarfaraz IqbalGaurab DuttaSarika GuptaGausal KhanSarita KumarGautam PandeySaritphat OrrapinGema Frühbeck MartínezSarma NandiniGemma PratSarvesh Pratap KashyapGene M. PestiSaša FrankGeng-Shi JengSasidharan SreenivasanGeoffrey MadeiraSaska IvanovaGeoffrey MitchellSaskia C. FlamentGeonsoo HaSathish K. R. PadiGeorg F. WeberSathya VelmuruganGeorge AnifandisSatona TanakaGeorge L. HinesSatoru MunakataGeorge N. KouvelosSatoshi KomatsuGeorge NotasSatyanarayan RoutrayGeorge PotamiasSatyavani KaliamurthiGeorgi As. GeorgievSaul Herranz-MartinGeorgia LevidouSaúl Martín RodríguezGeorgia TrakadaSaumik BasuGeorgia VrioniSaumik Saumik PanjaGeorgiana NitulescuSaumitra MukherjeeGeorgios BenetosSaurabh SinghGeorgios DryllisSaurav Kumar JhaGeorgios KoulaxouzidisSaurja DasguptaGeorgios P. PollakisSaverio MuscoliGeorgios VogiatzisSavina MannarinoGerasimos DarasSayaka MiuraGergely JakabSayanta BeraGergely RónaSayed Haidar Abbas RazaGergő JózsaScavello FrancescoGerhard KönigScott CoussensGerhild BornemannSebastian GłowińskiGermano CastelliSebastian PitaGhislain RocheleauSebastian SchloerGhulam MuhammadSebastian YuGiacomo BusoSebastian Ӧther-Gee PohlGiacomo Dal BelloSebastiano CiccoGiacomo RossettiniSebastien CalboGiada CrescioliSebok Kumar HalderGiada MagniSeca GandasecaGiamberto CasiniSedighe KarimzadehGian Marco GiuseppettiSegun OgundareGiancarlo CappelliniSeigo NishidaGiancarlo CondelloSeiichiro MitaniGianfranco SantovitoSelami Aykut TemizGianluca CasseseSelvaraj BarathiGianluca LuccheseSemra BilaçeroǧluGianluca NazzaroSenka MeštrovićGianluca RizzoSenta GrafGianmarco AbbadessaSenthil NagappanGianniantonio PetruzzelliSenthilnathan LakshmananGianpio SoriceSenwei TsaiGilberto CorsoSeok Hwee KooGilberto Gonzalez-AvalosSeon-Cheol ParkGionata FragomeniSeong Soo A. AnGiorgia AilunoSeong-Soo ChoiGiorgio BergaminiSerafeim PapadopoulosGiorgio CapuaniŞerban Vifor Gabriel BerteşteanuGiorgio LofreseSerena LucottiGiovanna Chaves CavalcanteSerena MontanariGiovanna CostanzoSerge V. NaugolnykhGiovanna Maddalena SquintaniSergei A. MoshkovskiiGiovanna MuscogiuriSergei KruglikGiovanna RomanoSergei Y. GrishinGiovanna SimonettiSergey AvdeevGiovanni Battista Levi SandriSergey KorchaginGiovanni ChiappettaSergey KozhukhovGiovanni DamianiSergey MoskvinGiovanni Francesco MarangiSergey SedykhGiovanni GalatiSerghei CovantevGiovanni LaviolaSergi MaicasGiovanni RibaudoSergio Galindo RodriguezGiovanni StelitanoSergio Gómez-RosalesGiovanni TarantinoSergio Soto SimentalGirum GetachewSerigo AcostaGisela BeutnerSeth FrietzeGiulia AbateSeung Kyu LeeGiulia BrindisiSevasti FilippidouGiulia FerrarazzoSeverino ZaraGiulia RadiSevilhan MennanGiuliana SolinasSeweta SrivastavaGiuliano FanelliSeyed Alireza SalamiGiuliano ZabucchiSeyed Amir MoosaviGiulio Cesare PassaliSeyed Arash AlawiGiuseppe CassoneSeyed Hossein HoseinifarGiuseppe D’AngeloSeyed Mohammad Ali Hosseini RadGiuseppe Di GiovanniSeyed Mohammad Nasir MousaviGiuseppe Emmanuele UmanaSeyedahmad SeyedalinaghiGiuseppe GrazianoSezer OkayGiuseppe GulloShaaban ElnesrGiuseppe LosurdoShagufta PerveenGiuseppe MancoShahana MajumderGiuseppe ManninoShahar ShellyGiuseppe MartelliShahbaz KhanGiuseppe MasciaShahid FarooqGiuseppe PanuccioShahin ImranGiuseppe ZaccaiShahrizan JamaludinGiuseppina AndreottiShahzad MunirGiuseppina BiondiShakur MohibiGiusi Irma ForteShalini DograGiusy TassoneShaloam DasariGlauco ChisciShambhu AryalGlen M. BoyleShamil SunyaevGlenda ErnstShams TabrezGonçalo GarciaShamshad KhanGonzalo VarelaShamsudheen Karuthedath VellarikkalGopal MaratheShane AustinGottfried J. PalmShangian TeeGoutam BanerjeeShankargouda PatilGraham SmithShao-Fang NieGrazia D'OnofrioShao-Jung HsuGraziele Freitas De BemShaolong SunGregor GrassShaoping LuGregory DoucendeSharath BalakrishnaGregory PoonSharifah Salmah Syed-HussainGrigorios TsigkasShariful IslamGrzegorz BartoszSharmistha BarthakurGrzegorz GrześkSharon MichelhaughGrzegorz KoniecznyShatha Ahmed AlduraywishGrzegorz KopecShazia RehmanGrzegorz LitwinienkoShazid Md. SharkerGrzegorz TrybekSheeraz AhmedGrzegorz WoźniakowskiSheila NielsenGrzegorz WysiadeckiSheila SpadaGrzegorz ZielińskiSheila VeroneseGuadalupe Alvarez HernanShekhar NeemaGuadalupe HerreraSheng ZhuGuadalupe Virginia Nevárez-MoorillónShenghuei LanGuangcai YinShengjing JiangGuanhong Wang‪Shengqian XiaGuda Mallikarjuna ReddyShengshuai ShanGuglielmo DurantiShensheng ChenGuglielmo ManticaShichao LvGuido MollShifat NowrinGuido ZavattaShigefumi OkamotoGuilherme FerreiraShigeki YamaguchGuillaume Anthony OdriShigemi SeoGuillaume MoissiardShigeyuki EsumiGuillaume StirnemannShiho NaitoGuillermo CeballosShihori TanabeGuillermo PlazaShimaa AmerGuillermo Santos-SánchezShin NishioGulam BahadurShin TakasawaGülşah Canakci AdigüzelShing-Hwa LiuGunawan Setia PrihandanaShingo KasamatsuGunjan PrakashShinichiro KashiwagiGuodong LuShin-Ichiro NomuraGuodong RaoShinichiro OgawaGuoping LiShinnkuang LinGuowei KimShintaro SukegawaGuoxun ChenShinya OhashiGuozheng WangShiran BordGustavo FernandesShireesh SrivastavaGustavo Rassier IsolanShiro AdachiGuy RostokerShiro SuetsuguGynendra Kumar RaiShiv VermaGyorgy KerekesShivalee DuduskarH. J. LacassieShivankar AgrawalH. Rob TaalShivraj YabajiHa Thi Thu TranShizhao LiHabib RahmanShogo HamaguchiHachung YoonShogo HaraguchiHacı SaglamShoji OuraHadeel M. M. Khalil BagyShowkat DarHadi RastinShraddha SinghHadith RastadShruti ShuklaHafiz Muhammad AsifShubai LiuHafiz-Muhammad-Umer FarooqiShubha Ranjan DuttaHai Long WangShuichi AsakawaHaishu LinShuji OginoHaiyang ChenShu-Jui KuoHaizhou ZhuShun-Fa YangHajar Fauzan AhmadShunichi IshiiHakan BozdoğanShun-Qing LiangHåkan N. PärssonShunsuke SaitoHala ShokrShuntaro TakahashiHalis SüleymanShuqiang WangHamada ElwanShusuke TodenHamdy ShaabanShuyu FuHamed Haddad KashaniShvetank BhattHamzeh AlipourShyi Jou ChenHan HanSiddharth NarayananHan XiaoSiddharth PahwaHanan A. Hosni MahmoudSiddharth Singh ChouhanHang YangSideris FotiadisHani AlhadramiSidney BeckerHani AlzaiatSiegfredo R. PaloyoHani N. NaseefSiew-Kee LowHan-Ki ParkSigit PrastowoHan-Mou TsaiSigrid A. LanghansHanna Evelina SidjabatSilvestro MesekaHanna KmitaSilvia Alberti-ViolettiHanna MoniuszkoSílvia Almeida CardosoHanne Christine BertramSilvia BistiHanneke Kwakkel-Van ErpSilvia CosimatoHanns MoshammerSilvia FilippiHanora AmroSilvia García-BujalanceHans G. DrexlerSilvia Gómez-JiménezHans-Christian SiebertSilvia MartuHansi WeissensteinerSilvia NovíoHans-Joachim SchüllerSilvia OndrašovičováHans-Michael TautenhahnSilvia PasquiniHans-Oliver RennekampffSilvia Regina Dias Medici SaldivaHans-Peter RusterholzSilvia RizzatoHany Ezzat KhalilSilvia RocchiccioliHanyan LiSilvia SauledaHanying ChenSilvia SvegliatiHao LiSilvia Valbuena-LópezHao-Ai ShuiSilvia VasiliuHaohua DengSime UkicHarald KrenzlinSimin SharifiHarald MatthesSimon D. Van HarenHarald PlattaSimon DuvalHarbinder SinghSimon SchoechlinHarish HandralSimon SieberHarminder SinghSimona AlibrandiHarpreet KaurSimona BoitoHarun AlbayrakSimona BungauHarun Egemen TolunaySimona CabibHarun ElcikSimona Delle MonacheHashim Talib HashimSimona GiordanengoHasintha WijesekaraSimona LaurinoHassan AlbarqiSimona LucioliHassan Al-KaragolySimona NicoaraHassan RanjbarSimona SolaHassan RasouliSimona VicasHatamian-Zarmi AshrafalsadatSimona ZaamiHatasu KobayashiSimona-Roxana GeorgescuHattaf KhalidSimone BattagliaHayato TsukamotoSimone BelliHazem ElkadySimone FattoriniHazem S. ElshafieSimone GrassiHazuki TamadaSimone MorselliHe YanSina M. HulkkonenHeather BogerSingh RajenderHeba ElgizawySirajudheen AnwarHéctor Beltrán-AlacreuSirasit SrinuanpanHéctor Capella-MonsonísSirijan SantajitHéctor González-De La TorreSirish KishoreHéctor M. Mora-MontesSirpa JalkanenHedwig E. DeubzerSkaidrius MiliauskasHee Jung KimSl KothariHee-Chul AhnSlađana MilardovićHee-Sung ChaeSlađana S. PopovićHefei ZhaoSlaven CrnkovicHeiko HeerklotzSlavica KvolikHeithem Ben AmaraSławomir KujawskiHelena BarrosoSlobodan ObradovicHelena H. ChowdhurySmijin Karthully SomanHelena Haque ChowdhurySmouni AbdelazizHelena MartynowiczSnjezana KastelanHelena MartyowiczSobia RanaHelmi Mohd Hadi PritamSocorro Retana-MarquezHelmut FickenscherSofia Bengoa LuoniHeloisa Nunes BordalloSohan JheetaHema RajaramSoibam Khogen SinghHemanth Kumar BoyinaSok Kuan WongHenrik C. BäckerSokol TrunguHenrik GalboSoleen GhafoorHenry LancashireSolomon OkbazghiHenry MunyandukiSolveig TosiHerbert Júnior DiasSomnath MukherjeeHermona SoreqSompong SansenyaHesam HashemianSonay AydınHiba Al-SayyedSonia Di GaetanoHicham El KhalilSonia FantoneHideaki OkajimaSonia RoncheyHideaki ShigaSonu Menachem Maimonides BhaskarHidekazkeu MoriyaSook Yee LiewHideyuki KinoshitaSoon-Hyuck LeeHimani PuniaSophia BarinovaHimanshu DeshwalSophia Ogechi EkeukuHimanshu GoelSophy Kathleen BarberHiroaki NekiSøren Rafael RafaelsenHiroaki TaniguchiSorin J. BrullHiroaki TobaSotaro FujiiHiroe KikuzakiSotirios FortisHirokazu TsukaharaSoumojit PalHiroki TeragawaSoumyadev SarkarHiroki ToyodaSouquan ZhangHiromi KurokawaSouvik KarHiroshi HasegawaSouzan Vergkizi-NikolakakiHiroshi ShibuyaSpaska StanilovaHirotada HiramaSpyros GkelisHirotaka NakashimaSquire BookerHiroyuki FukuiSree Deepthi MuthukrishnanHiroyuki KonnoSreeram UdayanHiroyuki KurataSrinath PashikantiHiroyuki SatohStacy SmithHiroyuki UetakeStarling EmeraldHitesh AgarwalStavros TryfonHo TsoiStefan BrockeHolger ErbStefan Cristian VesaHomero Reyes De La CruzŠtefan JanečekHomira BehbahaniStefan MihaicutaHon Lon TamStefan NaydenovHongliang HeStefan StefanovicHongping JinStefania LamponiHongqi LiStefania MerighiHongsheng ZhouStefania VitaleHong-Ting Victor LinStefanie WedepohlHongwei WangStefano AlcaroHongwu JingStefano CanosaHongyan SunStefano CivolaniHooi-Leng SerStefano DastoliHortensia Moreno-MacíasStefano LasaponaraHossam El-BeltagiStefano LuzzagoHossein AziziStefano Maria PriolaHossein SabouriStefano MartinaHoussem BoulebdStefano PaganoHouwen PanStefano SantabarbaraHovik SayadyanStefano VeraldiHowaida Abd-AllaStefano ZenobiHsi-Chieh LeeStefanos RoumeliotisHsin-Chien ChenStephan PayrHsin-Yao WangStephen H. PoorHsin-Yuan ChenStephen InbrajHsiu-Chen LinStephen J. FreelandHsiuying WangStephen PearceHsu-Heng YenSteven TrewickHuafeng WangSteven VanceHuapeng FengStig BrorsonHubert PakulaStyliani GeronikolouHueng-Chuen FanSu Datt LamHugo LujanSubhadip MukhopadhyayHugo Martínez-RojanoSubhamay PramanikHugo Pena-VerdealSubogh SinhaHugo SarmentoSudhan DebnathHugo Vélez-PérezSudip BhattacharyaHui ZhuSudip PaulHuibin LvSufang LiuHuifen ZhouSugato HajraHuixian HongSuhas KarkuteHun LeeSujoy KhanHung-Tse HuangSuman ChaudharyHung-Tsung WuSuman DuttaHung-Yi ChuangSuman SamantrayHüseyin CanSuman WattalHuseyin EdeSumera QasimHussain TahzibaSumira MalikHussain Yar KhanSumit VashisthHussein M. IsmailSun Young ParkHwayong LeeSundus JavedHye One KimSung-Ho HerHye Won JeongSunghyun YoonIain McgawSunil KarhadkarIan GriffinSunita MundaIbrahim Al-AshkarSunny Chi Lik AuIbrahim Celalettin HaznedaroğluSunny MalhotraIbrahim F. RehanSupanji SupanjiIgnacio JofréSupawadee PirataeIgnazio Gaspare VetranoSuprasanna PennaIgor A. ZolotukhinSupratim SenguptaIgor B. SivaevSupravat MukherjeeIgor DeynekoSuqin TangIgor Piotr TurkiewiczSuravajhala PrashanthIgor PottosinSuravoot YooyongwechIgor WaksmanSuresh KeshavamurthyIkabongo MukumbutaSuresh L. MIkhwan RinaldiSuresh V. ChinniIkuo KashiwakuraSuriya RehmanIlaria BaccaniSuruchi RoychoudhryIlaria CeccarelliSusanne RospleszczIlaria MarchioniSusanne VrtalaIlaria OttonelliSushil KumarIlaria RoatoSusumu MuroyaIleana CorvoSutherland MaciverIl-Hoon ChoSuzana OtaševićIlias S. TravlosSuzana ŽivkovićIlias SkeparniasSven MärdianIlknur SurSvetla Trifonova DanovaIlknur Sur ErdemSvetlana AidagulovaImad KansauSvetlana FaImadeldin ElfakiSvetlana MerenkovaIman MirmazloumSvetlana PolevovaIman RadSvetoslav TodorovImdad Ullah ZaidSwati TyagiImmaculata XessSy Duong-QuyImran ImranSyed A. QuadriInderbir SinghSyed Farhan AhmadIndu KohaarSyed Haris OmarInga CiprovičaSyed NaqviIngrid GarajováSyed Nasir Abbas BukhariIngrida BalnyteSyed Sayeed AhmadInigo Navarro-FernandezSylvère StörmannInna P. GladyshevaSylvia BoshraIntan Hakimah IsmailSylwia Bobis-WozowiczIoanna PoulopoulouSylwia TyrkalskaIoannis GeorgiouSyuhei BanIoannis LiampasSzymon SkoczyńskiIoannis NikolakakisT. J. HollingsworthIoannis ParaskevaidisT. P. KukinaIoannis RoussisTadashi FuruyamaIoannis VamvakarisTadashi ItoIoannis-Dimosthenis AdamakisTadeusz KowalskiIonel SamfiraTadeusz MuziolIonut DonoiuTae Young ShinIonut LuchianTae-Hwan KimIonut SchiopuTae-Jin KimIonut TudoranceaTae-Yoon LeeIordanis E. KonstantinidisTaha Koray ŞahınIosif MarincuTahereh EftekhariIouri E. BorissevitchTaihong LiuIqram HussainTakaaki HirotsuIraia Muñoa HoyosTakaaki SenbonmatsuIram MurtazaTakahiro NagatakeIrena GlažarTakahiro Watanabe-NakayamaIrena IlicTakahito MukaiIrena MilaniakTakamasa KinoshitaIrena NalepaTakashi NakakukiIrena RotermanTakashi NishimuraIrena SmagaTakashi OkijiIrene Claudia ViscontiTakashi TanikawaIrene P. CarvalhoTakaya HigakiIrfan A. RatherTakayuki HosonIrfan Ahmad BhatTakehiro IwataIrie Zoro BiTakemi OtsukiIrina GradinaruTakemichi FukasawaIrina IelciuTakeshi KakegawaIrina-Georgeta SufaruTakeshi KondohIriny AyoubTakuma InagawaIriny M. AyoubTakuya TadaIrshad AhmadTamara G. AmstislavskayaIsaak EffendyTamara PečenkováIsabel CasasTamás OllmannIsabel MarquesTamas ZakarIsabel Santos CardosoTancredo De SouzaIsabela Lourenço-TessuttiTaner SarIsabella Giulia FranzoiTania Mazzuca-SobczukIsabella PisaniTanja Grubić KezeleIsabella RussoTanja HofmannIsabella ZanellaTanja P. VasićIsabelle Grillier-VuissozTanmoy SanyalIsaiah PabuayonTanu AllenIsao OtsukaTanya StratevaI-Shiang TzengTao TangIslam A. KhalilTariq AliIslam AliTariq JavedIsmini E. PapageorgiouTariq MalikIsrael Casado-HernándezTarique HussainIstván KonczTaswar AhsanIstvan MihalyTatiana MinkinaI-Ta LeeTatiana SidiropoulouItamar GonçalvesTatjana Lj. MitrovićItay BarneaTatsuji MizukamiItzhak OrionTatsuo KandaIulia-Cristina BagiuTatyana PasechnikIuliana CotiTawin IemprideeIva KiracTayebeh KazemiIvan BratchenkoTeddy Weifa YangIván CarreraTejas KarhadkarIvan ČekerevacTerence L. KirleyIvan Gláucio Paulino-LimaTeresa ThielIvan KopitovićTeresita Paz-MaldonadoIvan M. SavicTerezia KiskovaIvan SavicTessa CarrauIvana KraljevicTetsumi TakahashiIvana MarekovićTetsuya KonishiIvana MilenkovićThais A. EnokiIvana NovakThanh-Tam HoIvana NovakovicThawatchai ChaijarasphongIvar ZekkerThayne KowalskiIvica GrkovićTheocharis ChatzistathisIvo Dumic-CuleTheodora KatsilaIzabela DudaTheodore Danso MarfoIzabela FeckaTheodore KarapanayiotidesIzabela Korona-GłowniakTheodoros KarantanosIzabela Małysz-CymborskaTheofani AntoniouIzabela RaganThirumurugan RathinasabapathyIzabela ZakrockaThirupathi RavulaIzhar Hyder QaziThiruvenkadan Aranganoor KannanIztok PrislanThomas BuhseJ. A. P. M. De LaatThomas CaspariJ. C. FloresThomas EbenhanJ. David PuettThomas FischerJ. Domínguez‐CheritThomas G. SmithJ. DumonceauxThomas HesselbergJ. Francis BorgioThomas M. TiefenboeckJ. Spencer JohnstonThomas MartinJ. Woods HalleyThomas NadererJacek LewandowiczThomas O. KragJacek MatysThomas Oliver MéröJacek TabarkiewiczThomas SciorJack J. W. A. Van LoonThuareag Monteiro Trindade Dos SantosJackson Victor De AraujoTia L. KauffmanJacob LisumaTiago NazarethJacob MashiahTianpeng ZhangJacob SchönTiara Bunga Mayang PermataJacques CabaretTiberiu Paul NeaguJae Ha LeeTiberius BalaesJae Kyu RyuTigran ChalikianJae-Hyung ParkTijana SubotičkiJagdeep RahulTilili BarhoumiJagesh K. TiwariTim HulsenJagodnik KathleenTim ReynoldsJagpreet SinghTimofey LebedevJai P. RaiTimothy A. CrombieJaime Bustos-MartínezTimothy EricksonJaime Pérez-VillanuevaTimothy N. C. WellsJaime RauTineke VanderhaeghenJair PutzkeTingwei ChangJakir HossainTitus CoorayJaksuma PongsetkulTiziana CollettiJakub DrozakTiziana GreggiJakub SawickiTjaard PijningJalal UddinTofazzal IslamJames B. WoolleyTolga ZamanJames C. L ChowTolstenkov OlegJames ChowTom J. SyerJames D. BlankenshipTomas NajerJames J. KohlerTomas SimurdaJames LeitchTomas TamošuitisJames P. BennettTomasz BogielJames P. RopaTomasz FrączykJames ShapiroTomasz GóreckiJames V. AquavellaTomasz KrystofiakJames X. HartmannTomasz MarjanskiJamil HussainTomasz PorażkoJamshad HussainTomasz PowrózekJan KucharskiTomasz RymarczykJan LukasTomasz TokarekJan MejzlikTomaž JagričJan NovákTommaso GrecoJan PaczesnyTommaso LombardiJan TunérTommaso NeriJan WinklerTomofumi NishinoJan ZabrzynskiTomoki NakamuraJan-Dirk RaguseTomoko SawadaJanet L. PaluhTomoya FujieJang-Hyuk YunToni ZeinounJanmejay ParhiTooru AndohJános G. FilepTorbjörn BackströmJanos PostaTorbjörn LedinJanusz J. PetkowskiTorsak TippairoteJanusz Konstanty-KalandykToru TaguchiJanusz KsiazykToshiaki MakinoJan-Willem TaanmanToshihiko TashimaJaroslav TóthToshihiro KushibikiJarosław DastychToshihiro TsurudaJarosław Jaszczur-NowickiToshio HattoriJarosław PanekTraian DumitraşcuJarosław TrȩbaczTresna DewiJasenka Gajdoš KljusurićTrevor J. KingJasenka WagnerTriantafyllos DidangelosJasmina IsakovićTrias ThireouJasna JurasovićTristan Chalvon-DemersayJason MillsTrond LøvdalJason T. ShearnTsair-Fwu LeeJason Thomas DuskeyTsan-Chi ChenJavad HeshmatiTsuchida SachioJavad JavidniaTsung-I HsuJavad TavakoliTsung-Jung LiangJavaid Akhter BhatTsutomu NakashimaJaved IqbalTsutomu NakazawaJavier CorralTsvetelina VelikovaJavier Fernández-RuizTuba Çiğdem OğuzoğluJavier Santos-AberturasTullio PiardiJawhar GharbiTuo MengJay TrivediTyng Yuan JangJayabalan NirmalTze-Huan LeiJayalakshmi VallamkonduTzyy-Leng HorngJayaraman SelvarajUdaiyappan JanakiramanJayesh J. AhireUehara TomokoJean Carlos BettoniUelinton PintoJean Franĉois MornexUfuk IlgenJean KerverUgur CavlakJean VanderpasUjjal BhawalJean-David MoreauUjjwal LayekJean-Denis TroadecUlf Jensen-KonderingJean-Luc MorelUlrikke VossJean-Marc RetrouveyUlyana ZubairovaJean-Marie ExbrayatUmberto MalapelleJean-Marie GrosboisUmberto RomeoJean-Paul DouliezÜmit ŞentürkJelena Nesovic OstojicUnai Perez-SautuJelena Osmanović BarilarÜner KolukisaogluJelena PrpicUriel ZapataJelena RoganovicUros BumbasirevicJelena VladićUros LjubobratovićJenni M. E. VirtanenUrszula JankiewiczJennifer HulingUsama Abd El-RazekJennifer SalinasUtkarsh JainJenniffer AnguloUtkarsh KohliJens AppelV. G. AbilashJens MüllerV. N. KompanichenkoJeong-Hwa WooV. RajinikanthJeong-Hyun KangVadanasundari VedarethinamJeong-Hyun LeeVadim GrubovJeremy P. WoodVadim KlimontovJeroen Van SchependomVahab Youssof ZadehJérôme DuisitVahid EslamiJérôme R. LechienVaia LambadiariJeronimo AuzmendiValentina DiniJerónimo González-BernalValentina Elisabetta BounousJerris HedgesValentina GambardellaJerzy DropińskiValentina NikolićJerzy SieńkoValentina ParacchiniJesse JupiterValentina PozziJessica GasparValentina RussoJesús María Gómez-SalineroValeria CapraJesús Martín-GilValeria Di DatoJesús Mateos MartínValeria Di OnofrioJesús Oliva-Pascual-VacaValeria LeoniJialei DuanValeria ToscanoJiali WangValeriu Marin ȘurlinJian XuValery V. LikhvantsevJian YangVander Monteiro Da ConceiçãoJian YeVanesa Soto-LeónJian ZhangVanessa D. CostaJianan ZhaoVangelis OikonomouJianbo TongVan-Giau VoJianchao LiVanja Čikeš-KečJianfeng WangVarun KumarJiangchuan ShenVasile DrugJianing MiVasile PotopJianjun WenVasileios SiokasJianliang SunVasiliki TsigkouJiann-Chu ChenVassilios PanoutsakopoulosJianqiang XuVed Prakash SinghJianzhou CuiVeera Csr ChittepuJianzhu LiuVeeranoot NissapatornJie LeVeerawat TeeranachaideekulJie WangVeerupaxagouda PatilJie YanVera SequeiraJie YangVeronica Elena TrombitasJien Jiun ChenVeronica SteriJihoon NahVesna M. CoricJihua HaoVesna RastijaJiin Cherng YenVicente Eliezer Vega-MurilloJijun HuangVicente Espinosa-SolisJill L. MaronVicente Marco-MolinaJimena MuciñoVicente UriosJin BaiVictor GarciaJing LiuVictor M. PulgarJingen ZhuVictor Manuel Mendoza-NuñezJinhe KangVictor MatheuJinkwan KimVictor PhaniJinli WangVictor R. AlekseevJinming HanVictoria RoshchinaJinshan XuViet-Khoa Tran-NguyenJiong ZhouVijay Kumar JidigamJiří BekVijay Rakesh Reddy SanikommuJiří PeteraVijaya Kumar NarneJitendra KumarVijayakumar PonnusamyJitesh KumarVijayakumar SukumaranJiwei ZhangVijayalakshmi VenkatesanJiyang WangVikas Kumar RoyJi-Yeong BaeVikas KushwahaJj LuszczkiVikrant RaiJoachim MüllerViktor KokhanJoakim StrömbergViktor SasiJoana Almeida PalhaViktor V. BrygadyrenkoJoana BarbosaViktor ZvarychJoana CruzViktoria ShcherbakovaJoana Margarida Magalhães FerreiraVilma KaškonienėJoanna FilipowskaVilma PinchiJoanna KazimierowiczVilma RatautaiteJoanna Kwiecińska-PirógVinay Kumar TyagiJoanna PieczyńskaVincent ReyesJoão MartinsVincent YeungJoao MouraVincenzo D'AntòJoão Paulo TribstVincenzo FiorentinoJoão RodriguesVincenzo NuzziJoaquim CarrerasVincenzo Romano SpicaJoaquim Murray Bustorff-SilvaVincenzo TufarelliJoaquin F. Mould-QuevedoVincenzo ZammutoJoaquín María CamposVineet Kumar KamalJobin Jose KattoorVineet MittalJocelyn ReaderVinod KumarJochen SchneiderVinod VermaJoe ZhuViola TamasiJoel StavansVioletta MacioszekJohanna NelknerViorica PatruleaJohannes GrevenVirendra YadavJohannes HoferVirginia Flores-MoralesJohari Yap AbdullahVirginia Rivero-BucetaJohn A. Albert St. CyrVirginia WotringJohn CoiaVirginija JankauskaiteJohn D. HorowitzVishal JaikaransinghJohn ElefteriadesVishal KhairnarJohn H. PyneVishnu RajputJohn HendryVisweswara PasupuletiJohn P. HulmeVitalie LisnicJohn RodgerVitalie VǎcǎraşJohn T. SchulzVivek P. ChavdaJohn Z. KissVivek PandeyJolanta Behnke-BorowczykVladimir A. LukhtanovJonathan BlackledgeVladimir BuchmanJonathan FusiVladimir DvoretskyJonathan S. CalvertVladimir DyakonovJongki ChoVladimir HeiskanenJoo Hee YoonVladimir JakovljevicJorddy Neves CruzVladimir KubyshkinJordi Maitas-GuiuVladimir SobolevJordi Martorell-MarugánVladimir SukhorukovJorge A. Roacho-PérezVladimir TesarJorge AntunesVladimir ValkovJorge H. SaavedraVladimir YeremeevJorge M. SilvaVladimir ZhemkovJose Antonio De Paz FernándezVolker BrözelJosé Antonio Morales-GonzálezVolodymyyr KravetsJose Antonio O'BrienVsevolod V. GurevichJose BanulsVu Dinh ThongJose C. AdsuarVui King Vincent-ChongJosé Caparros-MartinVytaute PeciulieneJosé ChiesWael A. Al-ZereiniJosé Cleiton Sousa Dos SantosWagdi SolimanJosé Dijair AntoninoWaheed AnwarJosé Luis Arce-DiegoWahid Ali Hamood AltowaytiJosé Luis Rodríguez GutiérrezWaleed Al-MarzooqiJosé Luis Vázquez NogueraWalid Ben RomdhaneJosé Luiz De Brito AlvesWalter Dario Cardona MayaJose M. GamonalesWalter J. FlapperJose M. MuletWalter Ribeiro JuniorJose MerodioWalter SchummJosé Pedraza ChaverriWan Faisham Wan IsmailJose PietriWan Nordiana RahmanJosé Rafael De AlmeidaWang JieJosé Ribamar Dos Santos Ferreira JúniorWan-Jin ChenJosé Rodriguez-RodriguezWannaporn IttiprasertJosef FinstererWaseem El-HuneidiJosefa Gonzalez-SantosWattanapong KurdthongmeeJosep M. Trigo-RodríguezWeber Andre AlbertoJoseph ByaruhangaWei GuoJoseph J. CrivelliWei KangJoseph MoranWei ShaoJosephraj VijayaraghavanWei WuJoshua A. DublandWei ZhuJosip A. BorovacWei-Biao LiaoJosipa BukicWei-Chieh HuangJozef SowinskiWei-Chieh LeeJualang Azlan GansauWeidong LiuJuan A. Fafián-LaboraWeihong LinJuan Antonio Martínez-TadeoWei-Hsiung YangJuan Antonio Valera-CaleroWei-Jen TingJuan J. BadimonWeiwei ZengJuan José Gonzalez GerezWeiyu LiJuan Luis Delgado-GallegosWen-Bin YangJuan M. GonzalezWen-Chieh SungJuan Manuel BussoWen-Der WangJuan Manuel CelliniWenfu TanJuan Manuel Marquez-RomeroWenhui GuJuan Mielgo-AyusoWenpeng ZhangJuan Miguel Soria GarciaWerner GrafJuan Pedro MartínezWerner HaselmayrJuan Pérez-FernándezWhitney A. BullockJuan Prado-OlivarezWilfried A. KuesJuan YangWilhelm MistiaenJude Juventus AweyaWilliam A. Petri Jr.Judit E. ŜponerWilliam DoerrlerJudith MihalyWilliam M. MeilJuho Antti MäkeläWilman CarrilloJulia A. ScottWinfried M. AmoakuJulia ErathWinglueng WongJulian Balanta-MeloWise YoungJulián Solís García Del PozoWissem MnifJuliana MoreiraWitold GertychJulie A. Carlsten ChristiansonWitold SkrzyńskiJulie ConstanzoWiwat ChancharoenthanaJulie Miller JonesWladimir Bocca Vieira De Rezende PintoJulie OldWojciech MagońJulie Pires Da SilvaWojciech PaslawskiJulien FreitagWolf Rainer AbrahamJulien PetillonWolfgang BernhardJulijus BogomolovasWolfgang HolnthonerJulio Calleja-GonzalezWolfgang NitschkeJulio CostaWolfgang TreimerJulio Plaza DíazWon KimJulio SoteloWon Kyong ChoJulio Vicente Figueroa-MillánWonchung LimJulius BogomolovasWoochun JunJumaa Salman ChiadWoon-Man KungJumpei SaitoXavier MontanéJun Hui SohXavier SonanichJun ImaiXi LouJun Jie Benjamin SengXiakun ChuJun KongXian-Fang MengJun MaXiang WangJun NishikawaXiangli ZhaoJun RenXiangtao LiJun SunXianxiu ChenJun ZhangXiao LinJunggon KimXiaodong MaJun-Ho LeeXiaodong YangJunhong WeiXiaofang ZhuJun-Ichi HanaiXiaolei TangJun-Ichi KawadaXiaolong FanJunichiro NakataXiaolu CambronneJunne-Ming SungXiaomao WuJunpei YamamotoXiaorong ZhangJunsoo ParkXiaoyan ZhengJunxuan MaXiaoyu ZhangJunya KusumotoXin LiJunyan TaoXin MuJurij LahXin YangJustin W. TaylorXin ZhangJustus G. GarwegXing LüJustyna MiłekXingbo ZhaoJustyna MiszczykXin-Luan WangJuwon OhXinmin LuJu-Yi ChenXin-Rong WangJyh-Fei LiaoXinxing ZhangJyotir Moy ChatterjeeXinyan ZhangK. C. KumawatXinzheng LiK. N. ChethanXisha WangK. S. V. Krishna RaoXiufen LiK. Singhachuit AchuitXu Dong ZhangKabirullah LutfyXuan MaKaio Cesar Chaboli AleviXuanang XuKai-Wen HsuXucheng HouKajal KhodamoradiXuehua XuKalman ImreXuelin ZhouKálmán ImreXuezhi LiKamal Hany HusseinXufei YangKamal SharmaXuming MaoKamil JurowskiYajun XieKamil KarolczakYamei LiKamil NelkeYamini KrishnanKana PuspitaYan CuiKang Min ParkYan HuangKang-Soo LeeYan LiuKangxu WangYana IgolkinaKannan ManiYang BoguangKannan SridharanYang LinKannappan ArunachalamYang Wen-ChiKanon JatuworaprukYangping LiKaraköse MustafaYangsean ChoiKaren L. PoseyYang-Teng FanKaren M. DoodyYangtian YiKaren P. FawleyYanis TamzaliKarim DorghamYannick ValléeKarin BlockYanping TianKarin MoellingYan-Ruide LiKarl-Philipp RommelYao-Ban ChanKaro MichaelianYaochun YuKarol BulskiYaoguang LiuKarolina Anna Wojtunik-KuleszaYaser GamallatKarolina DrężekYash GuptaKarolina GołąbekYasuko FujitaKarolina KupczyńskaYasukochi SatoshiKarolina OsowieckaYasumasa OkazakiKarthick RajanYasushi NishiiKarthika PrasadYasushige ShinguKarthikeyan SubburamuYen Chen TungKarthikeyan VenkatachalamYen-Chuan OuKarun ThongprajukaewYeongjae ChoiKatarzyna AugoffYeon-Ki KimKatarzyna Bąk-DrabikYheni DwiningsihKatarzyna GruszczynskaYhiya AmenKatarzyna HojanYi FangKatarzyna HolcmanYi ZhuKatarzyna KaczyńskaYide ZhangKatarzyna Kiec-KononowiczYifeng ChenKatarzyna KrzywickaYifeng QiKatarzyna KubiakYi-Hsien HsiehKatarzyna NucYi-Hung ChiangKatarzyna SkrypnikYijun PanKatarzyna StaszakYin LiKatarzyna WiniarskaYing ChenKate HsuYing JianxiKathleen D. CusickYing XuKathrin TyryshkinYinghuai ZhuKatia StefanantoniYinglun ZhanKatja SteigerYi-Qing YangKatsuki TsuchiyamaYi-Wei ChenKatsunori TeranishiYoann Michel GarnierKatsunori YoshidaYoann S. Le BretonKausar Hussain ShahYoel OhayonKausar Sadia FakhruddinYoko ItakuraKaushal Kishor RajakYolanda García-MesaKavindra Nath TiwariYong JinKavindra TiwariYong Weon YiKay Dietrich WagnerYong-Chan KimKazeem AdeyemiYongji ZengKazuhiko HashimotoYongjin ParkKazuhiko KotaniYongkui LiKazuhiro P. IzawaYongqiang ChenKazuhito SuzukiYoon-Yen YowKazuki M. MatsudaYoshiaki HasegawaKazumasa MatsumotoYoshifumi IwagamiKazushige OshitaYoshiharu KawaguchiKazuto TajiriYoshinari UeharaKazuyo IgawaYoshinobu KariyaKazuyuki TakaiYoshiro MatsuiKee-Lung ChangYoshiteru MorioKeisuke InomuraYosra A. HelmyKeita TamuraYosuke MatsuuraKeitaro SouYoung-Sik JungKeith A. MaggertYousef Ahmed FouadKellen ChenYousif SubhiKelly Da CostaYoussef KhamisKelly S. LevanoYoussef W. NaguibKen MasudaYouyoun MoonKenan BuldurunYozo NakazawaKengo SatoYu A. NasykhovaKenichi GotoYu Jin LimKenji FujiyoshiYu Kyung ChoKenji IkeharaYu LinKenji ShimazoeYu. V. IoniKenjiro BandowYuan LiuKenneth W. BerendzenYuan-Chuan ChenKenta NakaiYuan-Hao LiuKepa Ruiz-MirazoYuanhung WuKesawat Mahipal SinghYuanzhe LiKeshore BidaseeYu-Chan ChangKeun-Yeong JeongYuchin LienKe-Vin ChangYu-Ching LinKevin M. SullivanYueh ChoKevin P. JohnsonYuerou ZhangKevin R. HinkleYu-Hsiang KuanKezong QiYu-Hui WeiKhadija JavedYuichi SaitoKhaled AzizYuji NagatomoKhaled EmaraYuji ShimizuKhalid ElsaafienYujiao LuKhalil AhmedYuki NakamuraKhursheed ShiekhYuki YamamotoKiessoun KonatéYukihiko MomiyamaKimberly CockerhamYukinari KakizawaKimberly MccallYulia K. KomlevaKin Israel NotarteYulin RenKinga Grzech-LeśniakYumi Zuhanis Hasyun HashimKinga OstrowskaYumin DaiKiran LokhandeYun ShiKirill TkachenkoYungchieh TsaiKirill Vitalievich AzarinYunus Emre BeyhanKirsten LeisYuri RomanovKirsten VanniceYuriy MalyarKirthiram K. SivakumarYurong LiangKitiya VongkamjanYusuff OladosuKittisak SawanyawisuthYusuke YamamotoKiyohide UsamiYutian FengKiyonori KawasakiYu-Tse WuKiyotaka NemotoYuval KriegerKlára KosováYuwares MalilaKlaudia BanachYuya TakakuboKlaudia KocurekYves MechulamKlaus Edgar RothYvette MeißnerKlaus H. HoffmannZaara SarwarKlaus JungZachary BurtonKlaus KirchhofZachary F. BurtonKlaus-Dieter SchlüterZachary R. MccawKlearchos PsychogiosZachary S. ClaytonKlemen BohincZachary StrombergKlementina S. AvdeevaZaheel AhmedKohei UchimuraZahra GharariKoichi YoneyamaZahra ShahsavariKoji IchiharaZainab ShahbaziKoji IkegamiZaneta Czyznikowska-BalcerakKoji MikamiŻaneta Kimber-TrojnarKoji TamuraZarenezhad ElhamKoji YamatsuZarin ZainulKok-Min SeowZarqa NawazKolandaswamy AnbazhaganZarrin BasharatKollarigowda RavichandranZbigniew J. WitczakKomal KalaniZbigniew PutowskiKomivi DossaZbigniew RaszewskiKomuraiah MyakalaZdeňka BendováKomwit SurachatZdravka PoljakovičKonosuke NakajiZedong NieKonrad StępieńZeenat IqbalKonstantin Johannes ScholzZegbe Jorge A.Konstantin KozlovZehuan LiaoKonstantin KudrinZekuan YuKonstantinos DritsasZeliha SelamoğluKonstantinos SeretisŽeljka FiketKonstantinos SpanosŽeljka KneževićKoraljka Rajković-MolekŽeljko D. SavkovićKosuke MiyaiZengpeng LvKotapati Kasi ViswanathZenon PogorelićKouhei YamadaZesergio MeloKouichi SogaZexia GaoKouji MiyazakiZeynep Tacer CabaKoulla ParpaZhang LiuKourosh SheibaniZhaohai WangKoya OzawaZhaoqing ZengKresimir DolicZhe JinKrishna PatelZhe WangKrishna Prasad NeupaneZhe YangKristian LeisegangZhe ZhangKristóf Endre KárolyZhen QiuKrisztián KatonaZhen WangKrisztina BelaZhen YuanKrisztina PohóczkyZheng WangKrunoslav M. SvericZhenqiang YaoKrzysztof ChmielowiecZhibo MaKrzysztof GomułkaZhigang LiKrzysztof KuprenZhipeng HuKrzysztof LetachowiczZhixin DongKrzysztof MarciniecZhiyuan XuKrzysztof MyrdaZhongbing LuKrzysztof SitkoZhonggang FengKrzysztof StefaniakZhong-Ji QianKrzysztof WójcikZhongtian LiuKrzysztof ZdziarskiZhongwei LiuKsenija VelickovicZhuang WangKuanwei ShengZhumei RenKuan-Ying HsiehZifang ZhaoKubilay YıldırımZihao OuKuhan ChandruZihao YuanKui ZhangZinovia KefalopoulouKumánovics GáborZiwei LiuKumar AjeetZohreh JahanafroozKumar KatraguntaZoltan MagyarKumar SelvarajooZoltan SimenfalviKumiya SugiyamaZonghang ZhangKun LiZongkang ZhangKun LiuZongxiang TangKun WangZorana DobrijevićKundan MishraZrinka MihaljevićKundi YangZrinka ŠtritofKunio KawamuraZsolt S. VégváriKunka Mohanram RamkumarZsuzsanna KahanKuo-Chou ChiuZsuzsanna RecsanKuraku ShigehiroZubbair MalikKusumadewi YulitaZuoming XieKwang Yeon HwangZuzana FarkašováKwangmin KimZuzana VivodováKwang-Sig LeeZygmunt Warzecha


